# Results from the Survey of Antibiotic Resistance (SOAR) 2018–21 in Kuwait and the United Arab Emirates: data based on CLSI, EUCAST (dose-specific) and pharmacokinetic/pharmacodynamic (PK/PD) breakpoints

**DOI:** 10.1093/jac/dkaf284

**Published:** 2025-11-24

**Authors:** Didem Torumkuney, Eiman Mokaddas, Stefan Weber, Ian Morrissey, Nergis Keles, Anand Manoharan

**Affiliations:** Infectious Diseases Research Unit, GSK, London, UK; Microbiology Department, Faculty of Medicine, Kuwait University, Jabriya, Kuwait; Microbiology Laboratory & Reference Laboratory for Infectious Diseases, Purelab Sheikh Khalifa Medical City, Abu Dhabi, United Arab Emirates; Antimicrobial Focus Ltd., Sawbridgeworth, UK; Infectious Diseases Medical & Scientific Affairs, GSK, Levent Özdilek River Plaza No: 13 İç Kapı No: 61 Şişli, Beşiktaş/İstanbul 34330, Türkiye; Infectious Diseases Medical & Scientific Affairs, GSK, Mumbai, India

## Abstract

**Objectives:**

To determine antibiotic susceptibility of *Streptococcus pneumoniae* and *Haemophilus influenzae* from community-acquired respiratory tract infections in Kuwait and United Arab Emirates (UAE), collected in 2018–21.

**Methods:**

MICs determined by CLSI broth microdilution; susceptibility data interpreted using CLSI and EUCAST breakpoints.

**Results:**

A total of 49 *S. pneumoniae* and 79 *H. influenzae* and 49 *S. pneumoniae* and 34 *H. influenzae* were collected from Kuwait and UAE, respectively. Of the pneumococci, 26.5% (UAE) and 20.4% (Kuwait) were penicillin susceptible by CLSI oral/EUCAST low-dose breakpoints; by CLSI intravenous/EUCAST high-dose administration, 95.9% (UAE) and 85.7% (Kuwait) were susceptible. Similar susceptibility was observed to ceftriaxone, amoxicillin/clavulanic acid, amoxicillin and cefotaxime (CLSI: 73.5%–93.9%) but lower to other cephalosporins, tetracyclines, macrolides and trimethoprim/sulfamethoxazole. Fluoroquinolones were ≥98.0% susceptible (CLSI/EUCAST high-dose). Compared with CLSI, activity was similar by EUCAST high-dose or pharmacokinetic/pharmacodynamic (PK/PD) breakpoints with few exceptions. Of the *H. influenzae* isolates, 17.7% (Kuwait) and 8.8% (UAE) carried β-lactamases. Significant differences in *H. influenzae* susceptibility (CLSI) were found for amoxicillin/clavulanic acid (81.0% Kuwait/97.1% UAE) and fluoroquinolones (UAE: levofloxacin 88.2%, moxifloxacin 82.4%; Kuwait: both 98.7%). Susceptibility was overall >91.2% (CLSI) except for ampicillin (81.0% Kuwait) and trimethoprim/sulfamethoxazole (47.1% UAE/55.7% Kuwait). EUCAST susceptibility was similar, except for cefuroxime. PK/PD susceptibility was similar to CLSI, but lower for cefaclor, cefuroxime and macrolides.

**Conclusions:**

Pneumococci were susceptible to fluoroquinolones (>98%), amoxicillin and amoxicillin/clavulanic acid (>79%–94%), ceftriaxone and cefotaxime (>73%–94%). Susceptibility was lower to oral/low-dose penicillin (20%–27%), macrolides (33%–51%), tetracyclines (53%–59%) and trimethoprim/sulfamethoxazole (33%–61%). Susceptibility was generally >80% in *H. influenzae* except for trimethoprim/sulfamethoxazole (47%–56%).

## Introduction

Community-acquired respiratory tract infections (CA-RTIs) are an important world health problem that, if treated inappropriately or if patients have comorbidities, can result in hospitalization, with a third of patients with community-acquired pneumonia dying within 12 months of being discharged from the hospital.^1^ However, it is possible that comorbidities, age and various patient-related risk factors contributed to the mortality rate.^1^ Treatment of CA-RTIs is reliant on empiric antibiotic therapy through the use of national and international guidelines.^2^ A study of the use of antibiotics in CA-RTIs in primary health centres in Kuwait has shown that more than 90% of patients treated with antibiotics received inappropriate therapy.^3^ Furthermore, a public survey in Kuwait indicated that a third of those patients prescribed an antibiotic in the previous year had not completed the treatment, and over 25% of patients were self-medicating to treat non-bacterial infections.^4^ Such inappropriate antibiotic use can be associated with resistance development.^5^ There are few studies on antibiotic use in the United Arab Emirates (UAE), but a recent study has shown that the main use of antibiotics in the UAE was for community-acquired infections and that overall there was good compliance with current guidelines.^6^ In another study, it was observed that Kuwait and the UAE had similar levels of antibiotic use and community-acquired infection prevalence.^7^

*Streptococcus pneumoniae* and *Haemophilus influenzae* are the major bacteria associated with CA-RTIs.^8,9^ Both pathogens have shown increasing resistance to first-line antibiotics such as penicillin and ampicillin.^10,11^ As rates of resistance vary over time and from country to country, up-to-date surveillance data are essential to guide local antibiotic policies.^12^

The Survey of Antibiotic Resistance (SOAR), an international antibiotic resistance surveillance study, focuses on key respiratory pathogens that cause community-acquired infections and has been running since 2002 in the Middle East, Africa, Latin America, Asia-Pacific, Europe and the Commonwealth of Independent States countries.^13^ For this study, recent SOAR data from hospitals in Kuwait and the UAE have been analysed to provide a picture of the current state of antibiotic susceptibility of *S. pneumoniae* and *H. influenzae* associated with CA-RTIs in these two countries.

## Materials and methods

### Ethics

SOAR studies are not human subject studies. During the study, only microorganisms were examined.

### Collaborating centres

Isolates were provided between 2018 and 2021 from the Department of Medical Microbiology, University of Kuwait, Kuwait, and Sheikh Khalifa Medical City Hospital, Abu Dhabi, United Arab Emirates.

### Clinical isolates

Isolates of *H. influenzae* and *S. pneumoniae* from CA-RTIs (isolated within 48 h of hospitalization) were sent to a central laboratory (IHMA Europe, Monthey, Switzerland), where they were sub-cultured and re-identified. *H. influenzae* were re-identified by MALDI-TOF MS methodology, and *S. pneumoniae* identity was confirmed by optochin susceptibility and bile solubility. β-lactamase production was determined for each *H. influenzae* isolate by a chromogenic cephalosporin (nitrocefin) disc method. Duplicate isolates from the same patient were not accepted.

### Susceptibility testing

Isolates were evaluated for antibiotic susceptibility using broth microdilution methodology recommended by CLSI.^14^ Amoxicillin, amoxicillin/clavulanic acid (2:1 ratio as per CLSI guidelines^14,15^), amoxicillin/clavulanic acid (fixed clavulanic acid at 2 mg/L as per EUCAST guidelines^16^), azithromycin, cefaclor, cefdinir, cefixime, cefotaxime, cefpodoxime, ceftibuten, ceftriaxone, cefuroxime, clarithromycin, levofloxacin, moxifloxacin and trimethoprim/sulfamethoxazole (1:19 ratio) were tested against both respiratory pathogens. In addition, doxycycline, erythromycin and penicillin were tested against *S. pneumoniae* only, and ampicillin was tested against *H. influenzae* only. Susceptibility to the study drugs was calculated based on CLSI, EUCAST (dose-specific) and pharmacokinetic/pharmacodynamic (PK/PD) breakpoints.^15–17^ These breakpoints are shown in Tables 1–3. To fully assess antibiotics where high-dose therapies are available, susceptibility using EUCAST criteria was also calculated by combining percentage susceptible and susceptible, increased exposure into the susceptible category.^16^ The antibiotics with high-dose availability assessed in this way were as follows: amoxicillin (0.75–1 g oral, 3× daily), amoxicillin/clavulanic acid (0.875 g amoxicillin/0.125 g clavulanic acid oral, 3× daily), ampicillin (2 g IV, 4× daily), penicillin (2.4 g IV, 2 MU 4–6× daily), ceftriaxone (2 g IV, 2× daily), clarithromycin (0.5 g oral, 2× daily), erythromycin (1 g oral or IV, 4× daily), levofloxacin (0.75 g oral 2× daily, or 0.4 g IV 3× daily) and trimethoprim/sulfamethoxazole (0.24 g trimethoprim/1.2 g sulfamethoxazole oral or IV, 2× daily).^16^

**Table 1.** CLSI MIC breakpoints (mg/L) used for *S. pneumoniae* and *H. influenzae* isolates

	*S. pneumoniae*			*H. influenzae*		
Antimicrobial	S	I	R	S	I	R
Amoxicillin	≤2	4	≥8	—	—	—
Amoxicillin/clavulanic acid (2:1)^a^	≤2	4	≥8	≤2	4	≥8
Ampicillin	NT	NT	NT	≤1	2	≥4
Azithromycin	≤0.5	1	≥2	≤4	—	—
Cefaclor	≤1	2	≥4	≤8	16	≥32
Cefdinir	≤0.5	1	≥2	≤1	—	—
Cefixime	—	—	—	≤1	—	—
Cefotaxime (non-meningitis)	≤1	2	≥4	≤2	—	—
Cefpodoxime	≤0.5	1	≥2	≤2	—	—
Ceftibuten	—	—	—	≤2	—	—
Ceftriaxone (non-meningitis)	≤1	2	≥4	≤2	—	—
Cefuroxime^b^	≤1	2	≥4	≤4	8	≥16
Clarithromycin	≤0.25	0.5	≥1	≤8	16	≥32
Doxycycline	≤0.25	0.5	≥1	NT	NT	NT
Erythromycin	≤0.25	0.5	≥1	NT	NT	NT
Levofloxacin	≤2	4	≥8	≤2	—	—
Moxifloxacin	≤1	2	≥4	≤1	—	—
Penicillin (2.4 g, 2 MU × 4–6 IV)	≤2	4	≥8	NT	NT	NT
Penicillin (oral)	≤0.06	0.12–1	≥2	NT	NT	NT
Tetracycline	≤1	2	≥4	≤2	4	≥8
Trimethoprim/sulfamethoxazole^c^	≤0.5	1–2	≥4	≤0.5	1–2	≥4

—, not applicable; I, intermediate; NT, not tested; R, resistant; S, susceptible.

^a^Amoxicillin/clavulanic acid was tested at a 2:1 amoxicillin to clavulanic acid ratio; breakpoints are expressed as the amoxicillin component.

^b^Breakpoints used are for cefuroxime axetil (oral).

^c^Trimethoprim/sulfamethoxazole was tested at a 1:19 trimethoprim to sulfamethoxazole ratio; breakpoints are expressed as the trimethoprim component.

**Table 2.** EUCAST (dose-specific) MIC breakpoints (mg/L) used for *S. pneumoniae* and *H. influenzae* isolates

	*S. pneumoniae*		*H. influenzae*	
Antimicrobial^a^	S	R	S	R
Amoxicillin (0.5 g × 3 oral)	≤0.5	>1	≤0.001	>2
Amoxicillin (0.75–1 g × 3 oral)	≤1	>1	≤2	>2
Amoxicillin/clavulanic acid (0.5 g/0.125 g × 3 oral)^b^	≤0.5	>1	≤0.001	>2
Amoxicillin/clavulanic acid (0.875 g/0.125 g × 3 oral)^b^	≤1	>1	≤2	>2
Ampicillin (2 g × 3 IV)	NT	NT	≤1	>1
Ampicillin (2 g × 4 IV)	NT	NT	≤1	>1
Azithromycin	≤0.25	>0.5	—	—
Cefaclor	≤0.001	>0.5	—	—
Cefdinir	—	—	—	—
Cefixime	—	—	≤0.12	>0.12
Cefotaxime	≤0.5	>2	≤0.12	>0.12
Cefpodoxime	≤0.25	>0.5	≤0.25	>0.25
Ceftibuten	—	—	≤1	>1
Ceftriaxone (1 g × 1 IV)	≤0.5	>2	≤0.12	>0.12
Ceftriaxone (2 g × 2 IV)	≤2	>2	≤0.12	>0.12
Cefuroxime^c^	≤0.25	>0.5	≤0.001	>1
Clarithromycin (0.25 g × 2 oral)	≤0.25	>0.5	—	—
Clarithromycin (0.5 g × 2 oral)	≤0.5	>0.5	—	—
Doxycycline	≤1	>2	NT	NT
Erythromycin (0.5 g × 2–4 oral or 0.5 g × 2–4 IV)	≤0.25	>0.5	NT	NT
Erythromycin (1 g × 4 oral or 1 g × 4 IV)	≤0.5	>0.5	NT	NT
Levofloxacin (0.5 g × 2 oral or 0.4 g × 2 IV)	≤0.001	>2	≤0.06	>0.06
Levofloxacin (0.75 g × 2 oral or 0.4 g × 3 IV)	≤2	>2	≤0.06	>0.06
Moxifloxacin	≤0.5	>0.5	≤0.12	>0.12
Penicillin (0.6 g 1 MU × 4 IV)	≤0.06	>2	NT	NT
Penicillin (2.4 g 2 MU × 4–6 IV)	≤2	>2	NT	NT
Tetracycline	≤1	>2	≤2	>2
Trimethoprim/sulfamethoxazole (0.16 g/0.8 g × 2 oral or IV)^d^	≤1	>2	≤0.5	>1
Trimethoprim/sulfamethoxazole (0.24 g/1.2 g × 2 oral or IV)^d^	≤2	>2	≤1	>1

—, not applicable; NT, not tested; R, resistant; S, susceptible.

^a^Where available, susceptibility was assessed using EUCAST higher dosage breakpoints.

^b^Amoxicillin/clavulanic acid was tested at a fixed concentration of 2 mg/L; breakpoints are expressed as the amoxicillin component.

^c^Breakpoints used are for cefuroxime axetil (oral).

^d^Trimethoprim/sulfamethoxazole was tested at a 1:19 trimethoprim to sulfamethoxazole ratio; breakpoints are expressed as the trimethoprim component.

**Table 3.** PK/PD MIC breakpoints (mg/L) used for *S. pneumoniae* and *H. influenzae* isolates

	*S. pneumoniae* and *H. influenzae*
Antimicrobial	S only
Amoxicillin (1.5 g/day)^a^	≤2
Amoxicillin (4 g/day)^b^	≤4
Amoxicillin/clavulanic acid^a^ (1.75 g/0.25 g/day adults; 45 mg/6.4 mg/kg/day children)	≤2
Amoxicillin/clavulanic acid^b^ (4 g/0.25 g/day adults; 90 mg/6.4 mg/kg/day children)	≤4
Ampicillin	—
Azithromycin	≤0.12
Cefaclor	≤0.5
Cefdinir	≤0.25
Cefixime	≤1
Cefotaxime	—
Cefpodoxime	≤0.5
Ceftibuten	—
Ceftriaxone	≤1
Cefuroxime^c^	≤1
Clarithromycin	≤0.25
Doxycycline	≤0.25
Erythromycin	≤0.25
Levofloxacin	≤2
Moxifloxacin	≤1
Penicillin	—
Tetracycline	—
Trimethoprim/sulfamethoxazole^d^	≤0.5

—, not applicable; S, susceptible.

^a^Amoxicillin/clavulanic acid for low dose in adults/children.

^b^Amoxicillin/clavulanic acid for high dose in adults/children.

^c^Breakpoints used are for cefuroxime axetil (oral).

^d^Trimethoprim/sulfamethoxazole was tested at a 1:19 trimethoprim to sulfamethoxazole ratio; breakpoints are expressed as the trimethoprim component.

### Quality control and data analysis

Quality control strains *S. pneumoniae* ATCC 49619, *H. influenzae* ATCC 49247, *H. influenzae* ATCC 49766 and *Escherichia coli* ATCC 32518 were included on each day of testing. Results of susceptibility testing were only accepted if the results of the quality control strains were within the published acceptable range.

Differences in susceptibility between Kuwait and the UAE were assessed for statistical significance with Fisher's exact test using XLSTAT version 2023.1.1.1399. A *P* value of <0.05 was considered statistically significant. A similar statistical analysis was performed to compare the following:

1. Antibiotic susceptibility (using CLSI criteria) by penicillin susceptibility (*S. pneumoniae* only) for Kuwait and the UAE combined.
2. Antibiotic susceptibility (using CLSI criteria) of *S. pneumoniae* isolates from Kuwait during this study period (2018–21) versus SOAR data from Kuwait in 2015–17.^18^
3. Antibiotic susceptibility (using CLSI criteria) of *S. pneumoniae* and *H. influenzae* isolates from the UAE during this study period (2018–21) versus SOAR data from the UAE in 2011–13.^19^

## Results

### *S. pneumoniae* isolates

In total, 49 *S. pneumoniae* isolates were collected from Kuwait between 2018 and 2021. Most isolates came from sputum (*n* = 14, 28.6%) or blood (*n* = 14, 28.6%), with the remainder from endotracheal aspirate (*n* = 11, 22.4%), middle ear (*n* = 3, 6.1%), sinus (*n* = 1, 2.0%) and unidentified specimens (*n* = 6, 12.2%). Most isolates (*n* = 21, 42.9%) came from paediatric patients (aged ≤12 years), 19 (38.8%) were from adolescent and adult patients (aged 13–64 years) and nine (18.4%) were from elderly patients (aged ≥65 years).

Forty-nine *S. pneumoniae* isolates were collected from the UAE between 2018 and 2021. Virtually all isolates came from sputum (*n* = 40, 81.6%), with the remainder from blood and bronchoalveolar lavage (both *n* = 3, 6.1%), endotracheal aspirate (*n* = 2, 4.1%) and unidentified specimens (*n* = 1, 2.0%). An equal number of isolates (*n* = 20, 40.8%) came from paediatric patients (aged ≤12 years) and adolescent and adult patients (aged 13–64 years), with nine isolates (18.4%) from elderly patients (aged ≥65 years).

**Figure 1.**
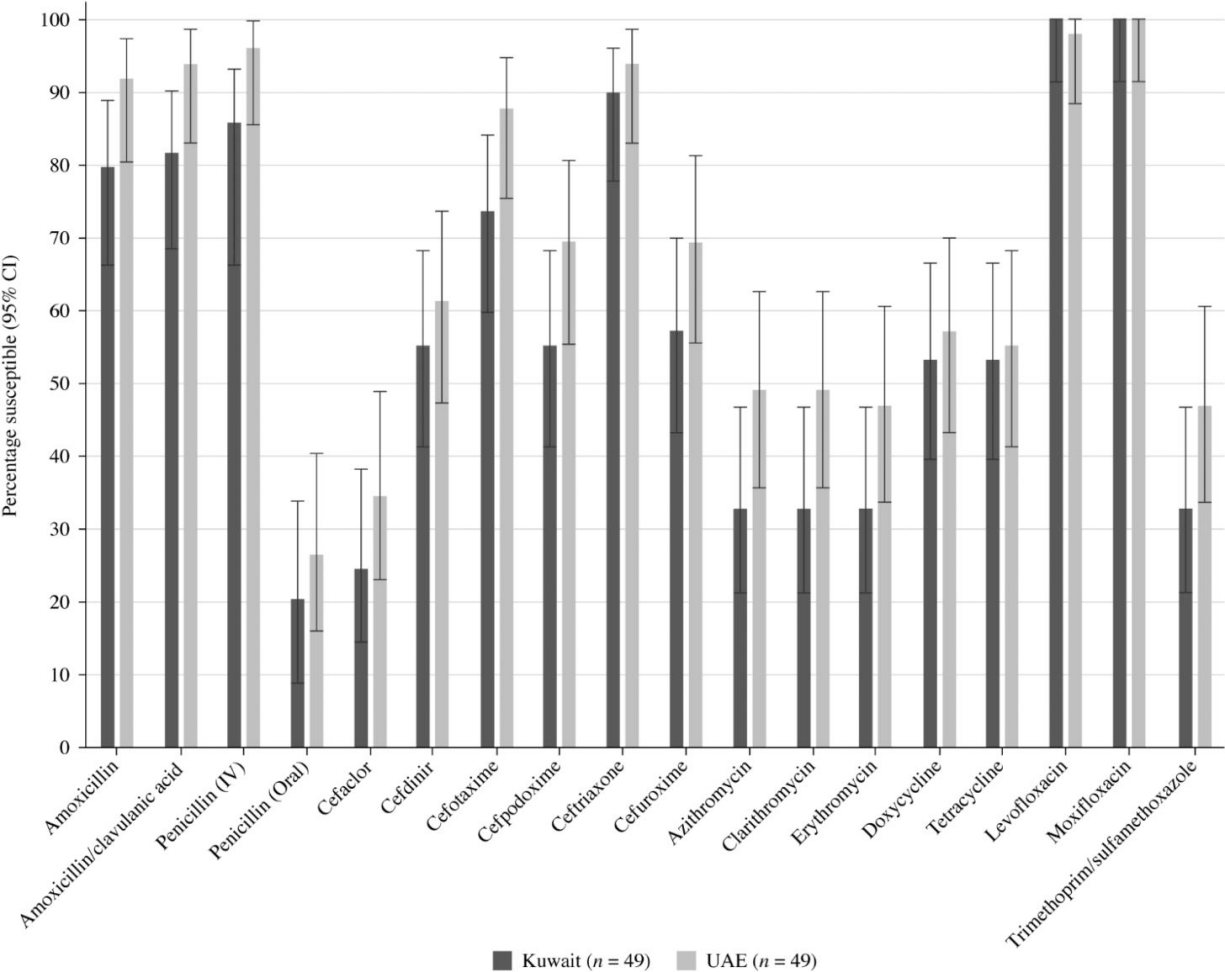
Antibiotic susceptibility rates (with 95% CI) of *S. pneumoniae* isolates from Kuwait (*n* = 49) and the UAE (*n* = 49) based on CLSI breakpoints. CI, confidence interval.

**Figure 2.**
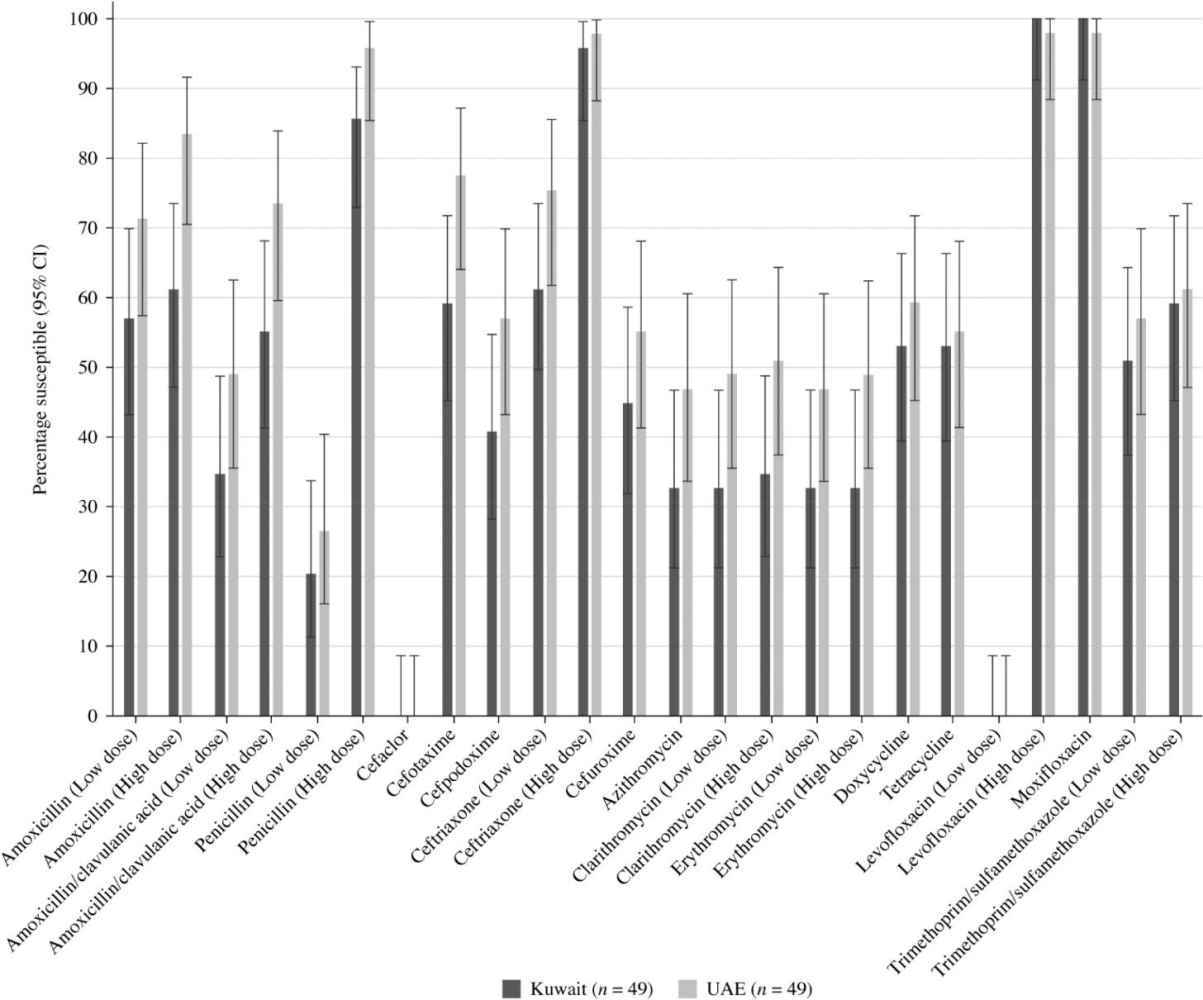
Antibiotic susceptibility rates (with 95% CI) of *S. pneumoniae* from Kuwait (*n* = 49) and the UAE (*n* = 49) based on EUCAST (dose-specific) breakpoints. CI, confidence interval.

Summary MIC, susceptibility and MIC distribution data for the 49 *S. pneumoniae* isolates from Kuwait and the 49 *S. pneumoniae* isolates from the UAE are shown in Tables 4–6 and S1 and S2 (available as Supplementary data at *JAC* Online). Comparative susceptibility data for both countries are shown in Figures 1 and 2.

**Table 4.** MIC and susceptibility data for *S. pneumoniae* isolates from Kuwait (*n* = 49) and the UAE (*n* = 49) using CLSI breakpoints

	Kuwait						UAE					
	MIC (mg/L)			CLSI susceptibility			MIC (mg/L)			CLSI susceptibility		
Antimicrobial	Range	50%	90%	%S	%I	%R	Range	50%	90%	%S	%I	%R
Amoxicillin	0.015–8	0.5	8	79.6	10.2	10.2	≤0.008–4	0.25	2	91.8	8.2	0
Amoxicillin/clavulanic acid (2:1)	0.015–8	0.5	8	81.6	8.2	10.2	≤0.008–4	0.25	2	93.9	6.1	0
Penicillin (2.4 g 2 MU × 4–6 IV)	0.015–4	0.5	4	85.7	14.3	0	≤0.008–8	0.25	2	95.9	2.0	2.0
Penicillin (oral)	0.015–4	0.5	4	20.4	42.9	36.7	≤0.008–8	0.25	2	26.5	57.1	16.3
Cefaclor	0.5 to >4	>4	>4	24.5	14.3	61.2	0.12 to >4	4	>4	34.7	8.2	57.1
Cefdinir	0.06 to >8	0.5	8	55.1	2.0	42.9	0.03 to >8	0.5	8	61.2	8.2	30.6
Cefixime	≤0.25 to >16	4	>16	—	—	—	≤0.25 to >16	2	16	—	—	—
Cefotaxime	0.015–4	0.25	2	73.5	22.4	4.1	≤0.008–4	0.25	2	87.8	8.2	4.1
Cefpodoxime	0.03 to >4	0.5	4	55.1	6.1	38.8	≤0.015 to >4	0.25	4	69.4	8.2	22.4
Ceftibuten	4 to >16	>16	>16	—	—	—	2 to >16	16	>16	—	—	—
Ceftriaxone	0.015–4	0.25	2	89.8	6.1	4.1	0.015–4	0.25	1	93.9	4.1	2.0
Cefuroxime	0.015 to >8	0.5	8	57.1	0	42.9	0.015 to >8	0.25	8	69.4	8.2	22.4
Azithromycin	0.12 to >16	16	>16	32.7	0	67.4	≤0.015 to >16	1	>16	49.0	4.1	46.9
Clarithromycin	0.03 to >16	8	>16	32.7	2.0	65.3	≤0.015 to >16	0.5	>16	49.0	2.0	49.0
Erythromycin	0.03 to >16	16	>16	32.7	0	67.3	≤0.015 to >16	2	>16	46.9	2.0	51.0
Doxycycline	0.06 to >4	0.12	>4	53.1	0	46.9	0.03 to >4	0.12	>4	57.1	2.0	40.8
Tetracycline	0.12 to >4	0.25	>4	53.1	0	46.9	0.12 to >4	0.25	>4	55.1	0	44.9
Levofloxacin	0.5–1	1	1	100	0	0	0.5 to >8	1	1	98.0	0	2.0
Moxifloxacin	0.06–0.25	0.12	0.12	100	0	0	≤0.03–1	0.12	0.25	100	0	0
Trimethoprim/sulfamethoxazole	0.12 to >8	1	8	32.7	26.5	40.8	≤0.06–8	1	8	46.9	14.3	38.8

—, not applicable; I, intermediate; R, resistant; S, susceptible.

**Table 5.** MIC and susceptibility data for *S. pneumoniae* isolates (*n* = 49) from Kuwait and the UAE (*n* = 49) using EUCAST (dose-specific) breakpoints

	Kuwait						UAE					
	MIC (mg/L)			EUCAST susceptibility			MIC (mg/L)			EUCAST susceptibility		
Antimicrobial	Range	50%	90%	%S	%I	%R	Range	50%	90%	%S	%I	%R
Amoxicillin (0.5 g × 3 oral)	0.015–8	0.5	8	57.1	4.1	38.8	≤0.008–4	0.25	2	71.4	12.2	16.3
Amoxicillin (0.75–1 g × 3 oral)	0.015–8	0.5	8	61.2	—	38.8	≤0.008–4	0.25	2	83.7	—	16.3
Amoxicillin/clavulanic acid (0.5 g/0.125 g × 3 oral)	0.03 to >8	1	>8	34.7	20.4	44.9	≤0.008 to >8	1	8	49.0	24.5	26.5
Amoxicillin/clavulanic acid (0.875 g/0.125 g × 3 oral)	0.03 to >8	1	>8	55.1	—	44.9	≤0.008 to >8	1	8	73.5	—	26.5
Penicillin (0.6 g 1 MU × 4 IV)	0.015–4	0.5	4	20.4	65.3	14.3	≤0.008–8	0.25	2	26.5	69.4	4.1
Penicillin (2.4 g 2 MU × 4–6 IV)	0.015–4	0.5	4	85.7	—	14.3	≤0.008–8	0.25	2	95.9	—	4.1
Cefaclor	0.5 to >4	>4	>4	0	12.2	87.8	0.12 to >4	4	>4	0	16.3	83.7
Cefdinir	0.06 to >8	0.5	8	—	—	—	0.03 to >8	0.5	8	—	—	—
Cefixime	≤0.25 to >16	4	>16	—	—	—	≤0.25 to >16	2	16	—	—	—
Cefotaxime	0.015–4	0.25	2	59.2	36.7	4.1	≤0.008–4	0.25	2	77.6	18.4	4.1
Cefpodoxime	0.03 to >4	0.5	4	40.8	14.3	44.9	≤0.015 to >4	0.25	4	57.1	12.2	30.6
Ceftibuten	4 to >16	>16	>16	—	—	—	2 to >16	16	>16	—	—	—
Ceftriaxone (1 g × 1 IV)	0.015–4	0.25	2	61.2	34.7	4.1	0.015–4	0.25	1	75.5	22.4	2.0
Ceftriaxone (2 g × 2 IV)	0.015–4	0.25	2	95.9	—	4.1	0.015–4	0.25	1	98.0	—	2.0
Cefuroxime	0.015 to >8	0.5	8	44.9	8.2	46.9	0.015 to >8	0.25	8	55.1	10.2	34.7
Azithromycin	0.12 to >16	16	>16	32.7	0	67.3	≤0.015 to >16	1	>16	46.9	2.0	51.0
Clarithromycin (0.25 g × 2 oral)	0.03 to >16	8	>16	32.7	2.0	65.3	≤0.015 to >16	0.5	>16	49.0	2.0	49.0
Clarithromycin (0.5 g × 2 oral)	0.03 to >16	8	>16	34.7	—	65.3	≤0.015 to >16	0.5	>16	51.0	—	49.0
Erythromycin (0.5 g × 2–4 oral or 0.5 g × 2–4 IV)	0.03 to >16	16	>16	32.7	0	67.3	≤0.015 to >16	2	>16	46.9	2.0	51.0
Erythromycin (1 g × 4 oral or 1 g × 4 IV)	0.03 to >16	16	>16	32.7	—	67.3	≤0.015 to >16	2	>16	49.0	—	51.0
Doxycycline	0.06 to >4	0.12	>4	53.1	8.2	38.8	0.03 to >4	0.12	>4	59.2	2.0	38.8
Tetracycline	0.12 to >4	0.25	>4	53.1	0	46.9	0.12 to >4	0.25	>4	55.1	0	44.9
Levofloxacin (0.5 g × 2 oral or 0.4 g × 2 IV)	0.5–1	1	1	0	100	0	0.5 to >8	1	1	0	98.0	2.0
Levofloxacin (0.75 g × 2 oral or 0.4 g × 3 IV)	0.5–1	1	1	100	—	0	0.5 to >8	1	1	98.0	—	2.0
Moxifloxacin	0.06–0.25	0.12	0.12	100	0	0	≤0.03–1	0.12	0.25	98.0	—	2.0
Trimethoprim/sulfamethoxazole (0.16 g/0.8 g × 2 oral or IV)	0.12 to >8	1	8	51.0	8.2	40.8	≤0.06–8	1	8	57.1	4.1	38.8
Trimethoprim/sulfamethoxazole (0.24 g/1.2 g × 2 oral or IV)	0.12 to >8	1	8	59.2	—	40.8	≤0.06–8	1	8	61.2	—	38.8

—, not applicable; I, susceptible, increased exposure; R, resistant; S, susceptible.

**Table 6.** MIC and susceptibility data for *S. pneumoniae* isolates (*n* = 49) from Kuwait and the UAE (*n* = 49) using PK/PD breakpoints

	Kuwait				UAE			
	MIC (mg/L)			PK/PD	MIC (mg/L)			PK/PD
Antimicrobial	Range	50%	90%	%S	Range	50%	90%	%S
Amoxicillin (1.5 g/day)	0.015–8	0.5	8	79.6	≤0.008–4	0.25	2	91.8
Amoxicillin (4 g/day)	0.015–8	0.5	8	89.8	≤0.008–4	0.25	2	100
Amoxicillin/clavulanic acid (1.75 g/0.25 g/day adults; 45 mg/6.4 mg/kg/day children)	0.015–8	0.5	8	81.6	≤0.008–4	0.25	2	93.9
Amoxicillin/clavulanic acid (4 g/0.25 /day adults; 90 mg/6.4 mg/kg/day children)	0.015–8	0.5	8	89.8	≤0.008–4	0.25	2	100
Penicillin	0.015–4	0.5	4	—	≤0.008–8	0.25	2	—
Cefaclor	0.5 to >4	>4	>4	12.2	0.12 to >4	4	>4	16.3
Cefdinir	0.06 to >8	0.5	8	30.6	0.03 to >8	0.5	8	44.9
Cefixime	≤0.25 to >16	4	>16	30.6	≤0.25 to >16	2	16	42.9
Cefotaxime	0.015–4	0.25	2	73.5	≤0.008–4	0.25	2	87.8
Cefpodoxime	0.03 to >4	0.5	4	55.1	≤0.015 to >4	0.25	4	77.6
Ceftibuten	4 to >16	>16	>16	—	2 to >16	16	>16	—
Ceftriaxone	0.015–4	0.25	2	89.8	0.015–4	0.25	1	93.9
Cefuroxime	0.015 to >8	0.5	8	57.1	0.015 to >8	0.25	8	69.4
Azithromycin	0.12 to >16	16	>16	18.4	≤0.015 to >16	1	>16	46.9
Clarithromycin	0.03 to >16	8	>16	32.7	≤0.015 to >16	0.5	>16	49.0
Erythromycin	0.03 to >16	16	>16	32.7	≤0.015 to >16	2	>16	46.9
Doxycycline	0.06 to >4	0.12	>4	53.1	0.03 to >4	0.12	>4	57.1
Tetracycline	0.12 to >4	0.25	>4	—	0.12 to >4	0.25	>4	—
Levofloxacin	0.5–1	1	1	100	0.5 to >8	1	1	98.0
Moxifloxacin	0.06–0.25	0.12	0.12	100	≤0.03–1	0.12	0.25	100
Trimethoprim/sulfamethoxazole	0.12 to >8	1	8	32.7	≤0.06–8	1	8	46.9

—, not applicable; PK/PD, pharmacokinetic/pharmacodynamic; S, susceptible.

### *S. pneumoniae* susceptibility

Antibiotic susceptibility by CLSI and EUCAST breakpoints was almost exclusively higher in the UAE compared with Kuwait. However, this difference was not statistically significant (Figures 1 and 2). Only 26.5% of pneumococci collected in the UAE and 20.4% from Kuwait were penicillin susceptible when CLSI oral or EUCAST low-dose IV breakpoints were applied. However, susceptibility to penicillin with EUCAST high-dose and CLSI IV breakpoints increased to 95.9% and 85.7% in the UAE and Kuwait, respectively. When following CLSI breakpoints, amoxicillin, amoxicillin/clavulanic acid and the third-generation cephalosporins ceftriaxone and cefotaxime showed similar activity, with susceptibility ranging from 87.8% to 93.9% in the UAE and from 73.5% to 89.8% in Kuwait. The third-generation cephalosporin cefdinir and second-generation cephalosporins cefpodoxime and cefuroxime were less active (61.2%–69.4% susceptible in the UAE and 55.1%–57.1% susceptible in Kuwait). The second-generation cephalosporin cefaclor was poorly active according to CLSI breakpoints (34.7% in the UAE and 24.5% in Kuwait) and PK/PD breakpoints (16.3% in the UAE and 12.2% in Kuwait). EUCAST breakpoints for amoxicillin and amoxicillin/clavulanic acid are lower than those from CLSI, showing reduced susceptibility compared with that obtained by CLSI breakpoints (49.0%–83.7% in the UAE and 34.7%–61.2% in Kuwait), even if higher doses are used. With higher dosing PK/PD breakpoints, full susceptibility for amoxicillin (4 g/day) and amoxicillin/clavulanic acid (4 g/0.25 g per day) was observed in UAE, and 89.8% susceptibility was observed in Kuwait. Cephalosporin susceptibility by EUCAST breakpoints is generally lower than that observed with CLSI breakpoints, especially for cefaclor, where no susceptible isolates were observed from either country. However, high-dose ceftriaxone retained 98.0% susceptibility in the UAE and 95.9% in Kuwait. Poor activity was observed for the macrolides (azithromycin, clarithromycin and erythromycin), tetracyclines (doxycycline and tetracycline) and trimethoprim/sulfamethoxazole by CLSI, EUCAST and PK/PD breakpoints (46.9%–61.2% susceptibility in the UAE and 18.4%–59.2% in Kuwait). Fluoroquinolone susceptibility was ≥98.0% in pneumococci from both countries using CLSI, EUCAST or PK/PD breakpoints, except for levofloxacin, where EUCAST high-dose was required (0% susceptible at low-dose breakpoint) (Tables 4–6 and S1 and S2 and Figures 1 and 2).

### Susceptibility of *S. pneumoniae* by penicillin resistance phenotype

**Figure 3.**
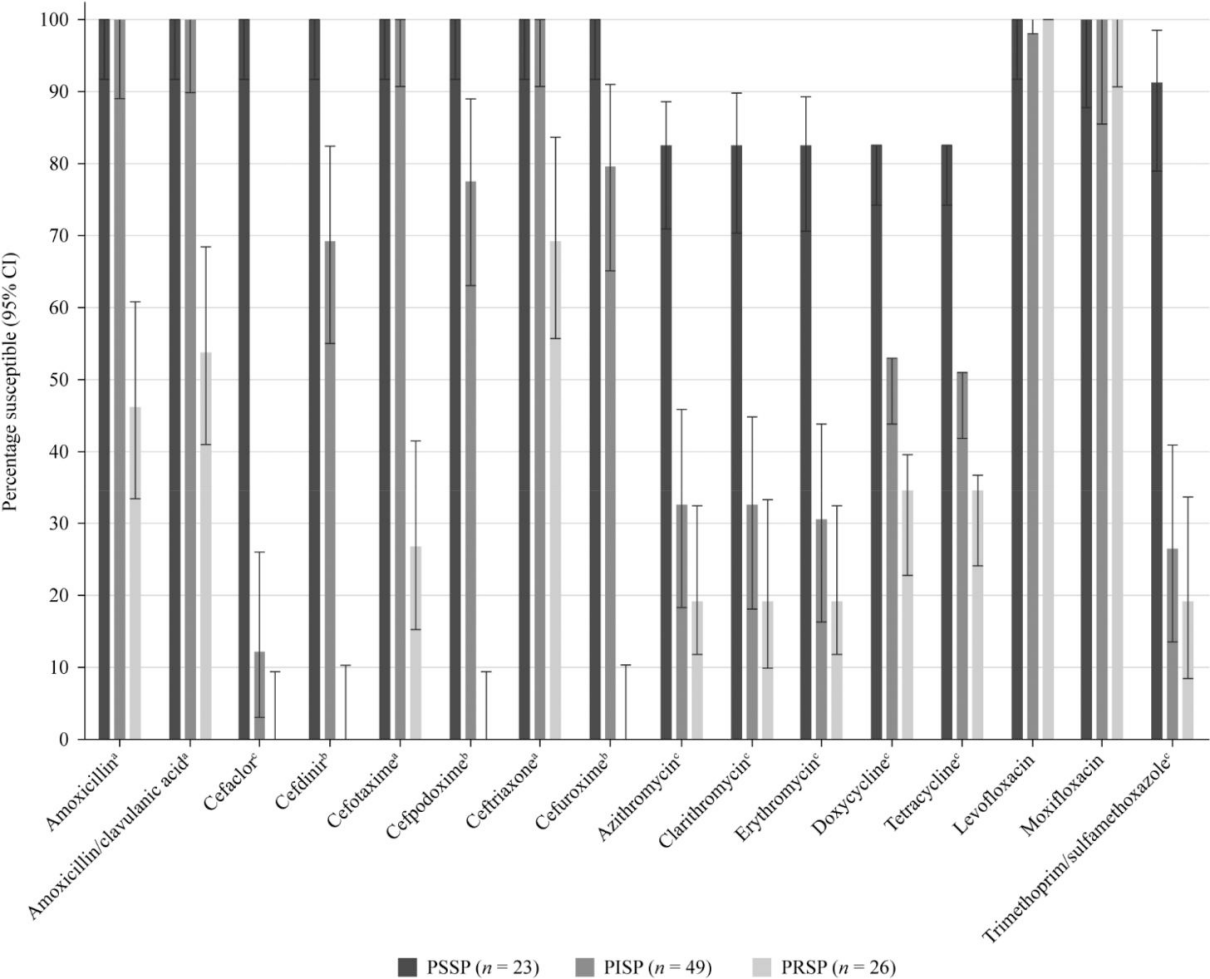
Susceptibility rates (with 95% CI) based on CLSI breakpoints for antibiotics against PSSP, PISP and PRSP from Kuwait and the UAE combined. Penicillin susceptibility categories are based on oral penicillin CLSI breakpoints. ^a^Susceptibility was significantly higher among PSSP than PRSP isolates (*P* < 0.005) and among PISP than PRSP isolates (*P* < 0.0001). ^b^Susceptibility was significantly higher among PSSP than PRSP isolates (*P* < 0.0001), among PSSP than PISP isolates (*P* < 0.01) and among PISP isolates than PRSP isolates (*P* < 0.0001). ^c^Susceptibility was significantly higher among PSSP than PRSP (*P* < 0.001) and among PISP than PRSP isolates (*P* < 0.0001). CI, confidence interval; PISP, penicillin-intermediate *S. pneumoniae*; PRSP, penicillin-resistant *S. pneumoniae*; PSSP, penicillin-susceptible *S. pneumoniae*.

An analysis of the activity of pneumococci from the UAE and Kuwait combined based on susceptibility to penicillin (CLSI oral breakpoints) was performed (Figure 3). Of the 98 combined *S. pneumoniae* isolates collected in both countries, 23 (23.5%) were penicillin-susceptible (PSSP), 49 (50.0%) were penicillin-intermediate (PISP) and 26 (26.5%) were penicillin-resistant (PRSP) according to CLSI oral breakpoints. All PSSP isolates were ≥82.6% susceptible to all antibiotics tested. PSSP isolates showed significantly higher (*P* < 0.005) susceptibility rates than PRSP isolates for all antibiotics, except for the fluoroquinolones, which showed ≥99.0% susceptibility irrespective of penicillin category. PSSP isolates also had significantly higher susceptibility than PISP isolates to cefaclor, cefdinir, cefpodoxime, cefuroxime, macrolides, tetracyclines and trimethoprim/sulfamethoxazole. Susceptibility rates of PISP isolates to the remaining antibiotics (amoxicillin, amoxicillin/clavulanic acid, cefotaxime, ceftriaxone and fluoroquinolones) were all ≥99.0%. Susceptibility rates of 0%–69.2% were observed against PRSP isolates for all antibiotics, except for levofloxacin and moxifloxacin (100% susceptible).

### Comparative susceptibility of *S. pneumoniae* collected in 2015–17 (Kuwait) and 2011–13 (UAE) with those from 2018–21

**Figure 4.**
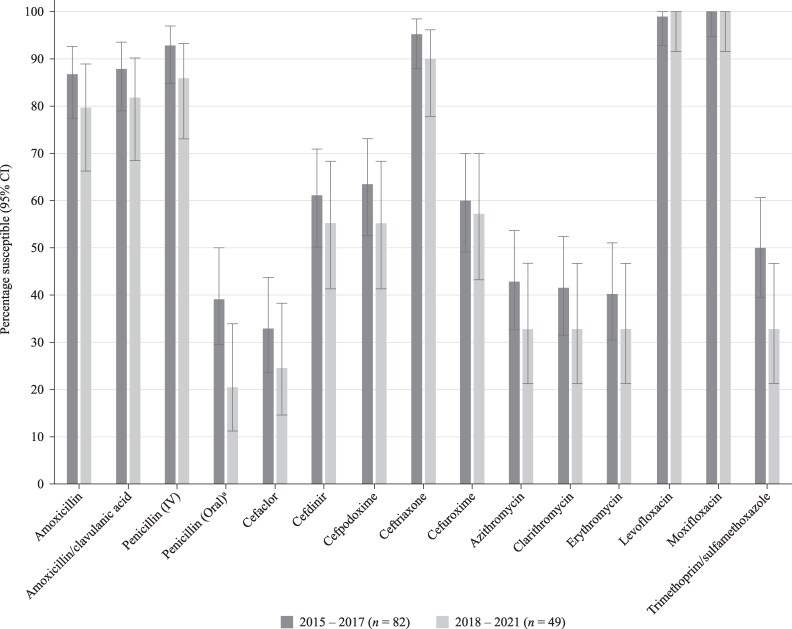
Comparison of antibiotic susceptibility rates of *S. pneumoniae* isolates from Kuwait collected in 2015–17 with isolates collected in 2018–21 (CLSI breakpoints). ^a^Susceptibility was significantly higher in 2015–17 than 2018–21 (*P* = 0.03). CI, confidence interval.

**Figure 5.**
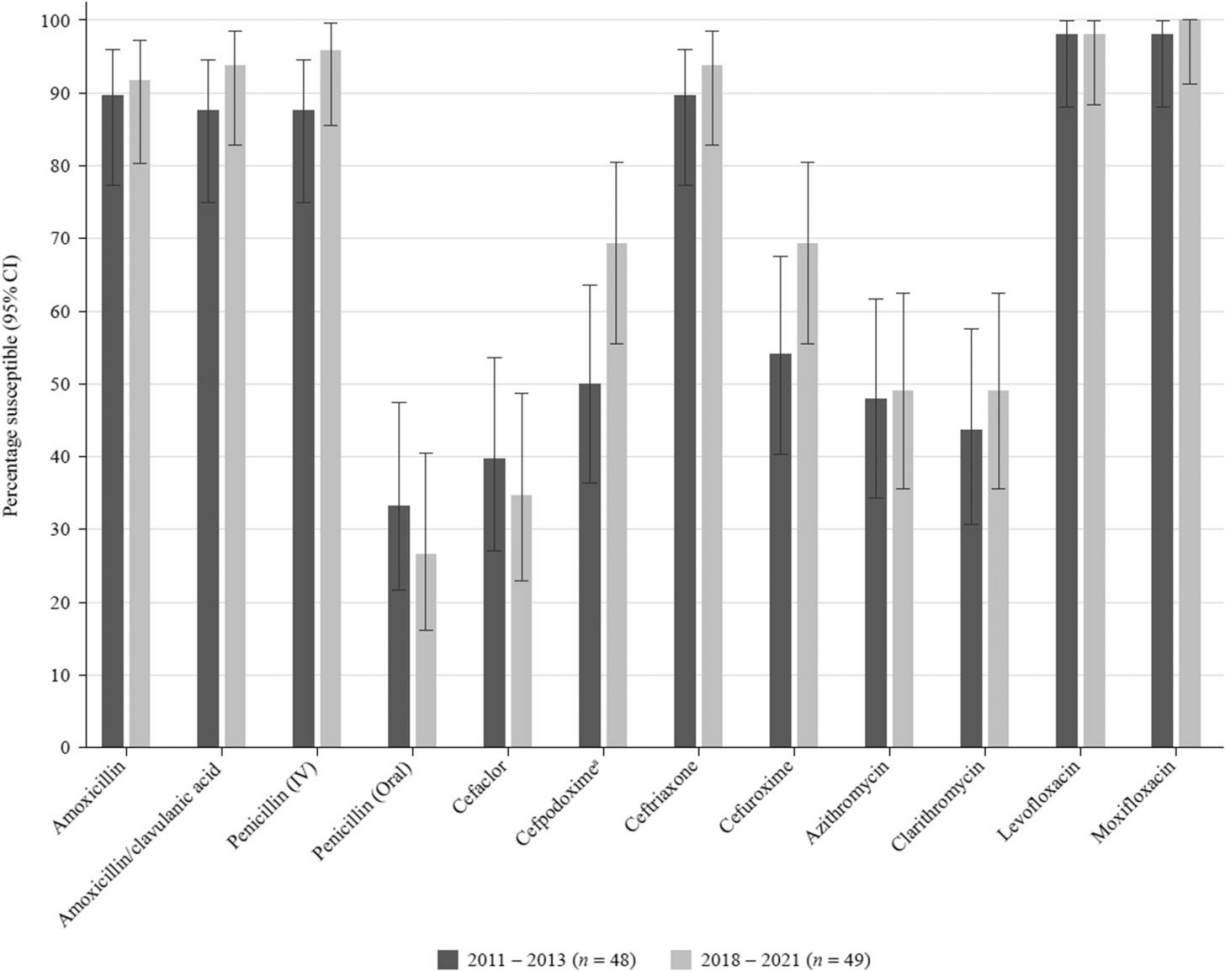
Comparison of antibiotic susceptibility rates of *S. pneumoniae* isolates from UAE collected in 2011–13 with isolates collected in 2018–21 (CLSI breakpoints). ^a^Susceptibility was significantly higher in 2018–21 than 2011–13 (*P* = 0.04). CI, confidence interval.

Data have previously been published from the SOAR surveillance for the period 2015–17 for pneumococci from Kuwait^18^ and were compared for mutually tested antibiotics with the current study (2018–21) using CLSI breakpoints (Figure 4). There was no significant change in susceptibility except for a decreased level of susceptibility to oral penicillin (39.0% versus 20.4%). However, the activity over either study period would be considered too low for therapeutic consideration. There was also a large numerical drop in susceptibility to trimethoprim/sulfamethoxazole (from 50.0% to 32.7%), but this was not statistically significant. Similarly, previously published SOAR data for *S. pneumoniae* from UAE in 2011–13^19^ were compared with data from the current study. As observed with Kuwait, there was little significant change in antibiotic susceptibility, with only cefpodoxime susceptibility significantly higher in 2018–21 compared with 2011–13; however, susceptibility remained below 70% (69.4%) (Figure 5).

### *H. influenzae* isolates

A total of 79 *H. influenzae* isolates were collected from Kuwait during 2018–21. Most isolates originated from sputum (*n* = 30, 38.0%). The remaining isolates were from endotracheal aspirate (*n* = 23, 29.1%), middle ear (*n* = 14, 17.7%), blood (*n* = 5, 6.3%), bronchoalveolar lavage (*n* = 4, 5.1%) and unidentified specimens (*n* = 3, 3.8%). Thirty-one (39.2%) of these isolates came from paediatric patients (aged ≤12 years), 29 (36.7%) isolates were from adolescent and adult patients (aged 13–64 years), and 19 (24.1%) isolates were from elderly patients (aged ≥65 years).

Thirty-four *H. influenzae* isolates were collected from the UAE during 2018–21. Most of these isolates originated from sputum (*n* = 24, 70.6%). The remainder were from blood or endotracheal aspirate (both *n* = 3, 8.8%), bronchoalveolar lavage (*n* = 1, 2.9%) or unidentified specimens (*n* = 3, 8.8%). Twenty (58.8%) of these isolates came from adolescent and adult patients (aged 13–64 years), eight (23.5%) isolates were from paediatric patients (aged ≤12 years) and six (17.6%) isolates were from elderly patients (aged ≥65 years).

**Figure 6.**
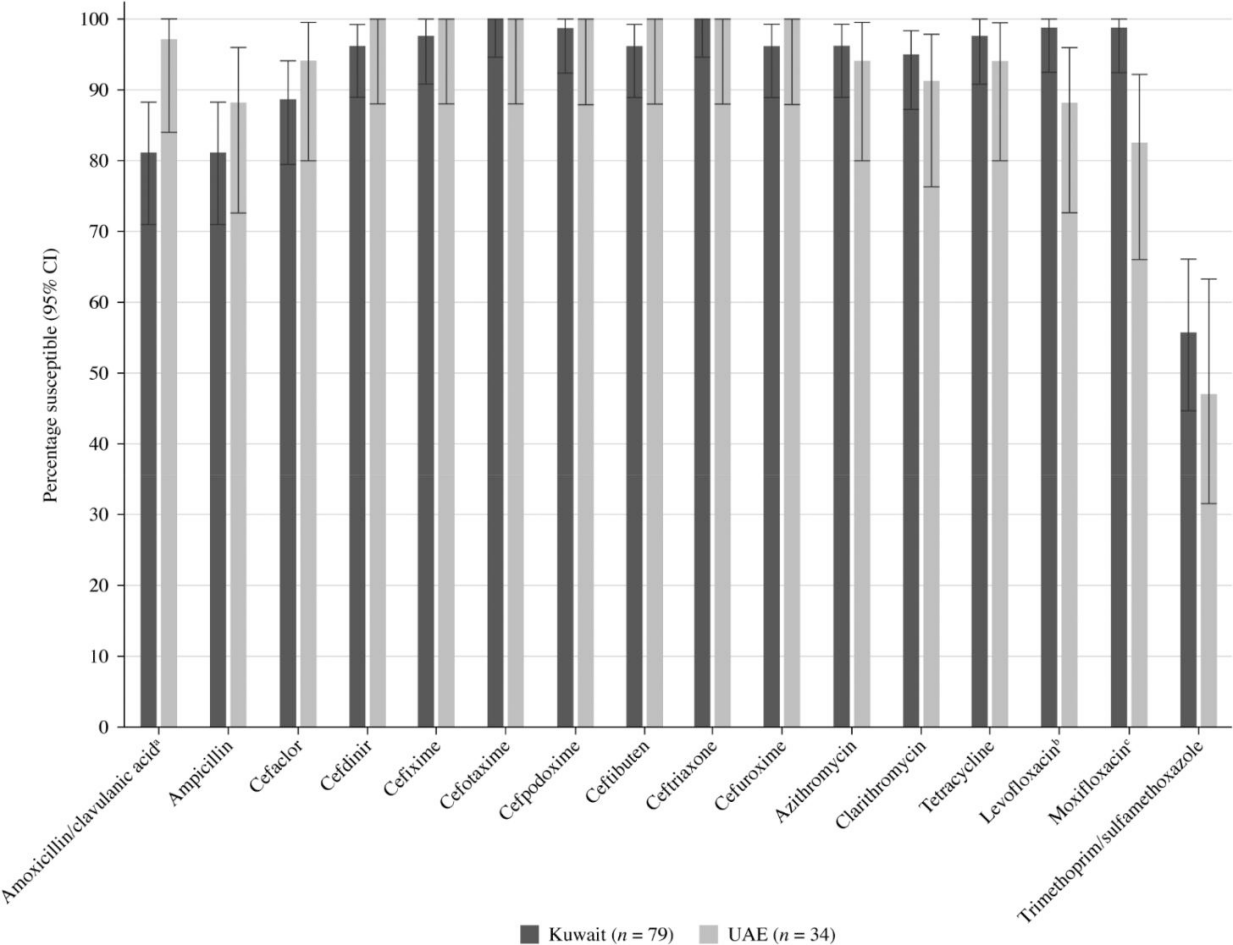
Antibiotic susceptibility rates of *H. influenzae* isolates from Kuwait (*n* = 79) and the UAE (*n* = 34) based on CLSI breakpoints. ^a^Susceptibility was significantly higher in the UAE than Kuwait (*P* = 0.04). ^b^Susceptibility was significantly lower in the UAE than Kuwait (*P* = 0.03). ^c^Susceptibility was significantly lower in the UAE than Kuwait (*P* = 0.003). CI, confidence interval.

**Figure 7.**
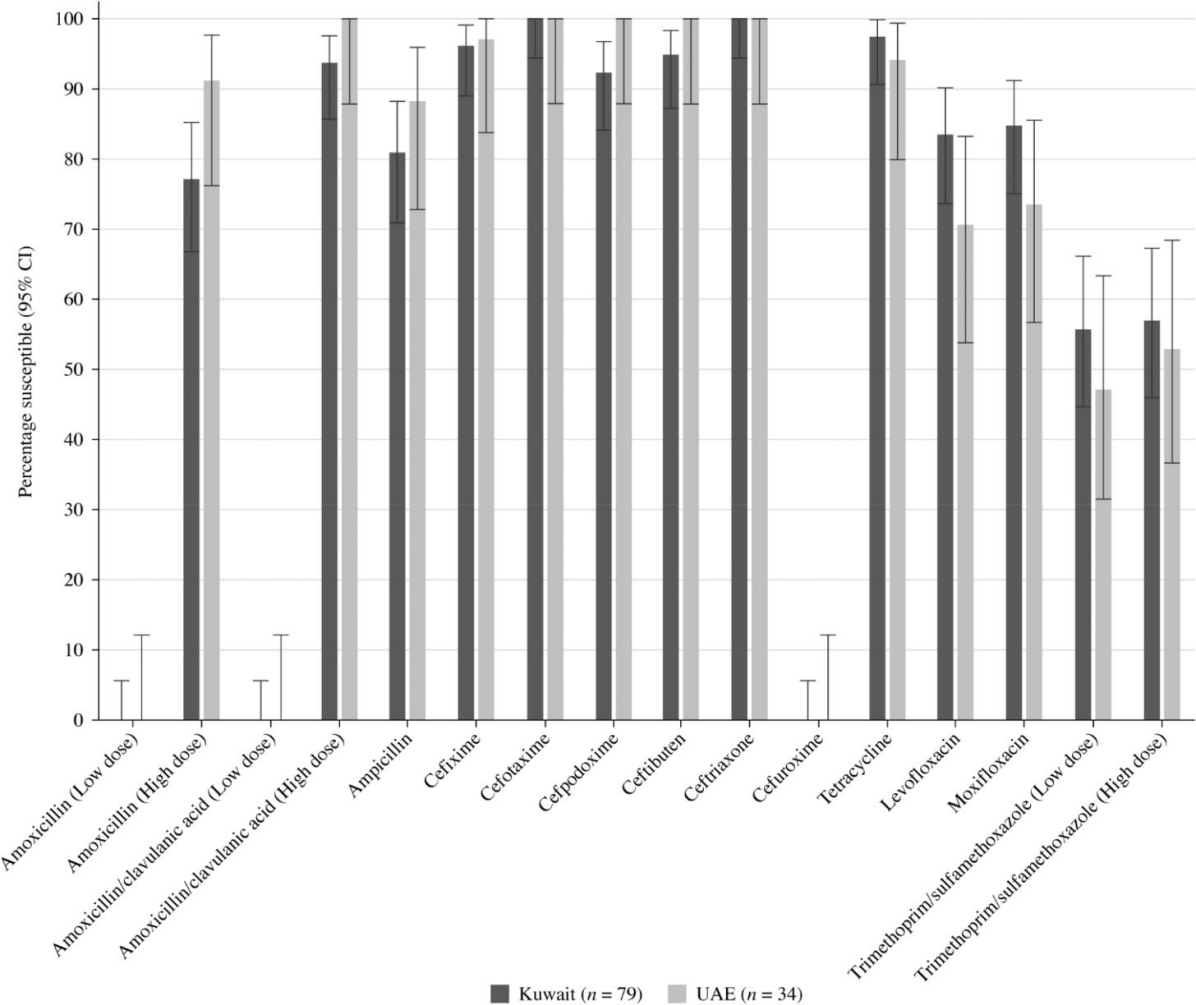
Antibiotic susceptibility rates of *H. influenzae* from Kuwait (*n* = 79) and the UAE (*n* = 34) based on EUCAST (dose-specific) breakpoints. CI, confidence interval.

Summary MIC, susceptibility and MIC distribution data for all 113 *H. influenzae* isolates are shown in Tables 7–9 and S3 and S4 and Figures 6 and 7.

**Table 7.** MIC and susceptibility data for *H. influenzae* isolates from Kuwait (*n* = 79) and the UAE (*n* = 34) using CLSI breakpoints

	Kuwait						UAE					
	MIC (mg/L)			CLSI susceptibility			MIC (mg/L)			CLSI susceptibility		
Antimicrobial	Range	50%	90%	%S	%I	%R	Range	50%	90%	%S	%I	%R
Amoxicillin	≤0.03 to >128	0.5	64	—	—	—	0.12–64	0.5	2	—	—	—
Ampicillin	≤0.03 to >128	0.5	64	81.0	1.3	17.7	0.06–64	0.25	2	88.2	2.9	8.8
Amoxicillin/clavulanic acid (2:1)	0.12–32	1	4	81.0	16.5	2.5	0.12–4	0.25	1	97.1	2.9	0
Cefaclor	≤0.25 to >32	2	16	88.6	7.6	3.8	0.5–32	2	4	94.1	2.9	2.9
Cefdinir	≤0.06 to >4	0.25	0.5	96.2	—	—	≤0.06–1	0.25	0.5	100	—	—
Cefixime	≤0.008–2	0.03	0.06	97.5	—	—	≤0.008–0.25	0.03	0.06	100	—	—
Cefotaxime	≤0.002–0.12	0.004	0.015	100	—	—	≤0.002–0.06	0.008	0.03	100	—	—
Cefpodoxime	≤0.015–4	0.06	0.25	98.7	—	—	≤0.015–0.25	0.06	0.25	100	—	—
Ceftibuten	≤0.008 to >4	0.06	0.25	96.2	—	—	≤0.008–1	0.06	0.25	100	—	—
Ceftriaxone	≤0.001–0.12	0.004	0.015	100	—	—	≤0.001–0.03	0.004	0.008	100	—	—
Cefuroxime	≤0.03 to >16	0.5	2	96.2	0	3.8	≤0.03–4	0.5	2	100	0	0
Azithromycin	≤0.12 to >8	1	2	96.2	—	—	≤0.12 to >8	1	2	94.1	—	—
Clarithromycin	≤0.25 to >32	4	8	94.9	1.3	3.8	≤0.25 to >32	4	8	91.2	5.9	2.9
Tetracycline	≤0.12–32	0.25	0.25	97.5	1.3	1.3	0.25–16	0.5	0.5	94.1	0	5.9
Levofloxacin	≤0.004–8	0.015	0.5	98.7	—	—	≤0.004 to >8	0.015	>8	88.2	—	—
Moxifloxacin	≤0.004–8	0.015	0.5	98.7	—	—	≤0.004 to >8	0.015	8	82.4	—	—
Trimethoprim/sulfamethoxazole	≤0.008 to >8	0.12	>8	55.7	5.1	39.2	≤0.008 to >8	1	>8	47.1	8.8	44.1

—, not applicable; I, intermediate; R, resistant; S, susceptible.

**Table 8.** MIC and susceptibility data for *H. influenzae* isolates from Kuwait (*n* = 79) and the UAE (*n* = 34) using EUCAST (dose-specific) breakpoints

	Kuwait						UAE					
	MIC (mg/L)			EUCAST susceptibility			MIC (mg/L)			EUCAST susceptibility		
Antimicrobial	Range	50%	90%	%S	%I	%R	Range	50%	90%	%S	%I	%R
Amoxicillin (0.5 g × 3 oral)	≤0.03 to >128	0.5	64	0	77.2	22.8	0.12–64	0.5	2	0	91.2	8.8
Amoxicillin (0.75–1 g × 3 oral)	≤0.03 to >128	0.5	64	77.2	—	22.8	0.12–64	0.5	2	91.2	—	8.8
Ampicillin	≤0.03 to >128	0.5	64	81.0	—	19.0	0.06–64	0.25	2	88.2	0	11.8
Amoxicillin/clavulanic acid (0.5 g/0.125 g × 3 oral)	≤0.03–16	0.5	2	0	93.7	6.3	0.06–2	0.25	1	0	100	0
Amoxicillin/clavulanic acid (0.875 g/0.125 g × 3 oral)	≤0.03–16	0.5	2	93.7	—	6.3	0.06–2	0.25	1	100	—	0
Cefaclor	≤0.25 to >32	2	16	—	—	—	0.5–32	2	4	—	—	—
Cefdinir	≤0.06 to >4	0.25	0.5	—	—	—	≤0.06–1	0.25	0.5	—	—	—
Cefixime	≤0.008–2	0.03	0.06	96.2	—	3.8	≤0.008–0.25	0.03	0.06	97.1	—	2.9
Cefotaxime	≤0.002–0.12	0.004	0.015	100	—	0	≤0.002–0.06	0.008	0.03	100	—	0
Cefpodoxime	≤0.015–4	0.06	0.25	92.4	—	7.6	≤0.015–0.25	0.06	0.25	100	—	0
Ceftibuten	≤0.008 to >4	0.06	0.25	94.9	—	5.1	≤0.008–1	0.06	0.25	100	—	0
Ceftriaxone	≤0.001–0.12	0.004	0.015	100	—	0	≤0.001–0.03	0.004	0.008	100	—	0
Cefuroxime	≤0.03 to >16	0.5	2	0	78.5	21.5	≤0.03–4	0.5	2	0	88.2	11.8
Azithromycin	≤0.12 to >8	1	2	—	—	—	≤0.12 to >8	1	2	—	—	—
Clarithromycin	≤0.25 to >32	4	8	—	—	—	≤0.25 to >32	4	8	—	—	—
Tetracycline	≤0.12–32	0.25	0.25	97.5	—	2.5	0.25–16	0.5	0.5	94.1	—	5.9
Levofloxacin	≤0.004–8	0.015	0.5	83.5	—	16.5	≤0.004 to >8	0.015	>8	70.6	—	29.4
Moxifloxacin	≤0.004–8	0.015	0.5	84.8	—	15.2	≤0.004 to >8	0.015	8	73.5	—	26.5
Trimethoprim/sulfamethoxazole (0.16 g/0.8 g × 2 oral or IV)	≤0.008 to >8	0.12	>8	55.7	1.3	43.0	≤0.008 to >8	1	>8	47.1	5.9	47.1
Trimethoprim/sulfamethoxazole (0.24 g/1.2 g × 2 oral or IV)	≤0.008 to >8	0.12	>8	57.0	—	43.0	≤0.008 to >8	1	>8	52.9	—	47.1

—, not applicable; I, susceptible, increased exposure; R, resistant; S, susceptible.

**Table 9.** MIC and susceptibility data for *H. influenzae* isolates from Kuwait (*n* = 79) and the UAE (*n* = 34) using PK/PD breakpoints

	Kuwait				UAE			
	MIC (mg/L)			PK/PD	MIC (mg/L)			PK/PD
Antimicrobial	Range	50%	90%	%S	Range	50%	90%	%S
Amoxicillin (1.5 g/day)	0.12–64	0.5	2	77.2	0.12–64	0.5	2	91.2
Amoxicillin (4 g/day)	0.12–64	0.5	2	82.3	0.12–64	0.5	2	91.2
Amoxicillin/clavulanic acid (1.75 g/0.25 g/day adults; 45 mg/6.4 mg/kg/day children)	0.12–4	0.25	1	81.0	0.12–4	0.25	1	97.1
Amoxicillin/clavulanic acid (4 g/0.25 g/day adults; 90 mg/6.4 mg/kg/day children)	0.12–4	0.25	1	97.5	0.12–4	0.25	1	100
Ampicillin	0.06–64	0.25	2	—	0.06–64	0.25	2	—
Cefaclor	0.5–32	2	4	8.9	0.5–32	2	4	8.8
Cefdinir	≤0.06–1	0.25	0.5	79.7	≤0.06–1	0.25	0.5	73.5
Cefixime	≤0.008–0.25	0.03	0.06	97.5	≤0.008–0.25	0.03	0.06	100
Cefotaxime	≤0.002–0.06	0.008	0.03	—	≤0.002–0.06	0.008	0.03	—
Cefpodoxime	≤0.015–0.25	0.06	0.25	93.7	≤0.015–0.25	0.06	0.25	100
Ceftibuten	≤0.008–1	0.06	0.25	—	≤0.008–1	0.06	0.25	—
Ceftriaxone	≤0.001–0.03	0.004	0.008	100	≤0.001–0.03	0.004	0.008	100
Cefuroxime	≤0.03–4	0.5	2	78.5	≤0.03–4	0.5	2	88.2
Azithromycin	≤0.12 to >8	1	2	5.1	≤0.12 to >8	1	2	8.8
Clarithromycin	≤0.25 to >32	4	8	1.3	≤0.25 to >32	4	8	5.9
Tetracycline	0.25–16	0.5	0.5	—	0.25–16	0.5	0.5	—
Levofloxacin	≤0.004 to >8	0.015	>8	98.7	≤0.004 to >8	0.015	>8	88.2
Moxifloxacin	≤0.004 to >8	0.015	8	98.7	≤0.004 to >8	0.015	8	82.4
Trimethoprim/sulfamethoxazole	≤0.008 to >8	1	>8	55.7	≤0.008 to >8	1	>8	47.1

—, not applicable; PK/PD, pharmacokinetic/pharmacodynamic; S, susceptible.

### *H. influenzae* susceptibility

Most isolates of *H. influenzae* were β-lactamase negative. However, 17.7% from Kuwait (14/79) and 8.8% from the UAE (3/34) carried β-lactamases. Within the β-lactamase negative populations, three isolates from Kuwait and one isolate from the UAE were β-lactamase negative ampicillin resistant (BLNAR) by EUCAST breakpoints (ampicillin MIC ≥2 mg/L) and two isolates from Kuwait by CLSI breakpoints (ampicillin MIC ≥4 mg/L). Two β-lactamase positive isolates from Kuwait were found to be ampicillin resistant (CLSI). In keeping with this β-lactamase and BLNAR status, 81.0% of isolates from Kuwait and 88.2% of isolates from the UAE were susceptible to ampicillin (CLSI or EUCAST breakpoints); however, this difference was not statistically significant. Amoxicillin breakpoints were not provided by CLSI, but susceptibility using EUCAST breakpoints at high-dose or PK/PD high-dose was 77.2% and 82.3%, respectively, in Kuwait and 91.2% in the UAE. Although numerically distinct, this difference was also not statistically significant. At EUCAST low-dose amoxicillin, no isolate from either country would be considered susceptible to amoxicillin. Susceptibility of isolates to amoxicillin/clavulanic acid by CLSI breakpoints was 81.0% in Kuwait and 97.1% in the UAE, which is statistically significant (*P* = 0.04). However, amoxicillin/clavulanic acid susceptibility using high-dose EUCAST breakpoints was 93.7% in Kuwait and 100% in the UAE. The higher PK/PD (4 g/0.25 g per day) dose increased amoxicillin/clavulanic acid susceptibility to 97.5% in Kuwait and 100% in the UAE. Susceptibility in Kuwait for most cephalosporins was ≥96.2% according to CLSI breakpoints, except for cefaclor, where 88.6% susceptibility was observed. Even lower cefaclor susceptibility of 8.9% was observed using PK/PD breakpoints. Similar results were seen for *H. influenzae* from Kuwait with EUCAST breakpoints (although these are not provided for cefaclor or cefdinir), except for cefuroxime (96.2% by CLSI versus 0% by EUCAST). Cephalosporin susceptibility in *H. influenzae* from the UAE was higher or the same as that in Kuwait in most cases, although the difference was not statistically significant. Macrolide breakpoints were not provided by EUCAST against *H. influenzae*; however, when applying CLSI breakpoints, azithromycin susceptibility was 96.2% in Kuwait and 94.1% in the UAE, and clarithromycin susceptibility was 94.9% in Kuwait and 91.2% in the UAE. In contrast, most isolates are considered macrolide non-susceptible according to PK/PD breakpoints. Tetracycline susceptibility was 97.5% in Kuwait and 94.1% in the UAE by both breakpoint standards, but fluoroquinolone susceptibility using CLSI breakpoints was 98.7% (levofloxacin) and 98.7% (moxifloxacin) in Kuwait and 88.2% (levofloxacin) and 82.4% (moxifloxacin) in the UAE. These differences in fluoroquinolone susceptibility were statistically significant. Fluoroquinolone susceptibility using EUCAST breakpoints was 83.5% (levofloxacin) and 84.8% (moxifloxacin) in Kuwait and 70.6% (levofloxacin) and 73.5% (moxifloxacin) in the UAE, but these differences were not statistically significant. Trimethoprim/sulfamethoxazole showed the weakest activity, with susceptibility of 55.7% and 47.1% by CLSI, PK/PD and low-dose EUCAST breakpoints in Kuwait and the UAE, respectively, and 57.0% and 52.9% susceptible by high-dose EUCAST breakpoints in Kuwait and the UAE, respectively (Tables 7–9 and S3 and S4 and Figures 6 and 7).

### Comparative susceptibility of *H. influenzae* collected in 2011–13 and 2018–21

**Figure 8.**
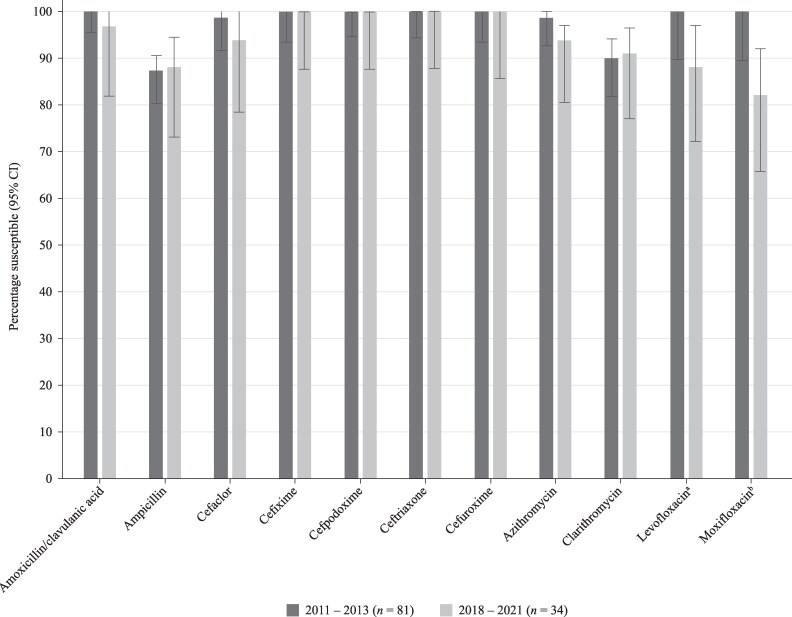
Comparison of antibiotic susceptibility rates of *H. influenzae* isolates from the UAE collected in 2011–13 with isolates collected in 2018–21 (CLSI breakpoints). ^a^Susceptibility was significantly higher in 2011–13 than 2018–21 (*P* = 0.007). ^b^Susceptibility was significantly higher in 2011–13 than 2018–21 (*P* = 0.0005). CI, confidence interval.

Data have previously been published from the SOAR surveillance for the period 2011–13 for *H. influenzae* from the UAE and were compared for mutually tested antibiotics with the current study (2018–21) using CLSI breakpoints (Figure 8). Susceptibility of *H. influenzae* from UAE to most antimicrobials has changed little since 2011–13, with no statistical difference between the years except for fluoroquinolones, where levofloxacin and moxifloxacin susceptibility was 100% in 2011–13^19^ but 88.2% and 82.4%, respectively, in the current study.

## Discussion

SOAR is an ongoing global surveillance study focusing on the two main CA-RTI pathogens, *S. pneumoniae* and *H. influenzae*, that have been monitored in numerous countries since 2002. Kuwait has participated in recent years, but the UAE has only previously participated in the periods from 2004 to 2006 and 2011 to 2013. The data presented here are an analysis of the antibiotic susceptibility of *S. pneumoniae* and *H. influenzae* isolates collected in Kuwait and the UAE between 2018 and 2021.

Although not statistically significant, the antibiotic susceptibility of *S. pneumoniae* in the UAE was generally higher than that in Kuwait. Data from both countries confirm that oral penicillin or low-dose IV penicillin is not an appropriate treatment regimen for CA-RTIs, as data using EUCAST low-dose IV or CLSI oral breakpoints showed only 26.5% susceptibility in the UAE and 20.4% in Kuwait. There has been a significant reduction in oral penicillin susceptibility in Kuwait since the last SOAR surveillance in 2015–17 (from 39.0% to 20.4% susceptible).^18,20^ A prior study of *S. pneumoniae* from Kuwait between 1997 and 2007 showed, on average, 56.0% penicillin susceptibility (using oxacillin discs equivalent to CLSI oral penicillin MIC breakpoint), with a low of 51.3% in 1997–2001 and a high of 61.3% in 2002–05.^21^ Another study from 2001 to 2004 indicated 37% susceptibility to oral penicillin.^22^ Although there is some study-to-study variability, it is clear that oral penicillin susceptibility in Kuwait has dropped dramatically since 1997. Data from the UAE showed 33.3% oral penicillin susceptibility during SOAR 2011–13,^19^ supporting this apparent trend. Data from the current study support the concept that higher-dose IV penicillin is a better therapeutic option, with susceptibility at 95.9% in the UAE and 85.7% in Kuwait. CLSI breakpoints indicate a similar level of susceptibility for amoxicillin, amoxicillin/clavulanic acid, ceftriaxone and cefotaxime, from 87.8% to 93.9% in the UAE and from 73.5% to 89.8% in Kuwait. Cefdinir, cefpodoxime, cefuroxime and cefaclor were less active than the other cephalosporins. EUCAST high-dose ceftriaxone susceptibility was 98.0% in the UAE and 95.9% in Kuwait; however, EUCAST breakpoints, even at the higher dose, indicate lower susceptibility for amoxicillin, amoxicillin/clavulanic acid and cefotaxime (≤83.7% in UAE and ≤61.2% in Kuwait) than those indicated by CLSI. Higher dosing with PK/PD breakpoints increased susceptibility to amoxicillin and amoxicillin/clavulanic acid. Susceptibility following CLSI guidelines, EUCAST guidelines and PK/PD breakpoints indicated poor activity for macrolides, tetracyclines and trimethoprim/sulfamethoxazole but good activity for fluoroquinolones against *S. pneumoniae* from both countries. Furthermore, apart from fluoroquinolones, there was a clear association between low penicillin susceptibility and low susceptibility to other antibiotics, as observed with the SOAR 2015–17 data from Kuwait.^18^ Data from the ATLAS database for 2014–19 indicated a similar level of low susceptibility to erythromycin and higher susceptibility to amoxicillin/clavulanic acid and ceftriaxone, with highest susceptibility to fluoroquinolones, as seen in the current study; however, a higher level of oral penicillin susceptibility but not IV penicillin was observed.^20^

In the current study, we compared the susceptibility of pneumococci using CLSI breakpoints for isolates previously collected in 2015–17 from Kuwait and 2011–13 from UAE with susceptibility from the current study (2018–21). There was no significant difference in susceptibility between the two study periods in Kuwait, except for reduced oral penicillin susceptibility (as noted above). There was a large difference in susceptibility to trimethoprim/sulfamethoxazole over the two study periods, where it reduced from 50.0% to 32.7%, but this was not statistically significant (*P* = 0.06). Similarly, there was little change in susceptibility in UAE over the two study periods, except for cefpodoxime where susceptibility increased significantly from 50.0% to 69.4%. However, this increase in susceptibility is most likely not high enough to consider cefpodoxime as a therapeutic option for pneumococci.

*H. influenzae* isolates from Kuwait and the UAE were mainly β-lactamase negative (82.3% and 91.2%, respectively), with three BLNAR isolates from Kuwait and one from the UAE using EUCAST breakpoints and two from Kuwait according to CLSI breakpoints. Ampicillin susceptibility was overall in accordance with β-lactamase prevalence (81.0% in Kuwait and 88.2% in the UAE by EUCAST breakpoints). When comparing the countries, any numerical difference in susceptibility between Kuwait and the UAE was not statistically significant using EUCAST breakpoints; this was also true using CLSI breakpoints in most cases. Amoxicillin/clavulanic acid was one exception where statistically significant higher susceptibility was observed in *H. influenzae* isolates from the UAE (97.1%) compared with those from Kuwait (81.0%). The other exception was the fluoroquinolones, where significantly higher susceptibility was observed in Kuwait (98.7% to moxifloxacin and levofloxacin) compared with the UAE (82.4% to moxifloxacin and 88.2% to levofloxacin). Apart from trimethoprim/sulfamethoxazole (47.1% susceptible in the UAE and 55.7% susceptible in Kuwait), most other antibiotics were very effective by CLSI breakpoints (≥81.0% in Kuwait and ≥82.4% in the UAE). Some EUCAST breakpoints are much lower than their CLSI counterparts, producing reduced susceptibility as seen with cefuroxime (96.2% and 100% by CLSI in Kuwait and the UAE versus 0% by EUCAST in both countries) and, to a lesser extent, with the fluoroquinolones: moxifloxacin (98.7% by CLSI versus 84.8% by EUCAST in Kuwait and 82.4% by CLSI versus 73.5% by EUCAST in the UAE) and levofloxacin (98.7% by CLSI versus 83.5% by EUCAST in Kuwait and 88.2% by CLSI versus 70.6% by EUCAST in the UAE). Data from the ATLAS surveillance interactive database for *H. influenzae* collected from 2015 to 2017 showed that levofloxacin susceptibility by EUCAST and CLSI breakpoints in Kuwait was 89.2% and 96.4% overall, confirming the results of the current study.^23^ Data from the same ATLAS study confirmed high antimicrobial agent susceptibility in *H. influenzae* from Kuwait between 2015 and 2017 using CLSI breakpoints.^20^

In the current study, we compared the susceptibility of *H. influenzae* using CLSI breakpoints for isolates previously collected in 2011–13 from the UAE with susceptibility from the current study (2018–21). Such data were not available for Kuwait. There was no significant difference in susceptibility between the two study periods, except for a significant reduction in fluoroquinolone susceptibility (from 100% to 88.2% for levofloxacin and 100% to 82.4% for moxifloxacin).

Differences in susceptibility between blood and non-blood isolates were evaluated globally; the data were presented at ESCMID Global 2025^24,25^ but are not included in this article. Susceptibility rates for *S. pneumoniae* and *H. influenzae* were generally similar between blood and non-blood isolates. However, reduced susceptibility was observed in some non-blood isolates compared with blood isolates, particularly: penicillin (oral), trimethoprim/sulfamethoxazole and second-generation cephalosporins (*S. pneumoniae*) and aminopenicillins, trimethoprim/sulfamethoxazole and levofloxacin (*H. influenzae*).

A limitation of both the current and previous SOAR studies is the potential bias in reported susceptibility rates due to antibiotic exposure before sample collection, which may have favoured the growth of resistant strains. Given its real-world focus, the SOAR programme assessed resistance patterns as encountered by clinicians worldwide. Therefore, inclusion of patients with prior antibiotic exposure was permitted, consistent with common clinical practice of initiating empirical antibiotic therapy before sample collection.

To conclude, low antimicrobial susceptibility rates of *S. pneumoniae* isolates (<50%) from Kuwait to many antibiotics were observed, and in the UAE, reduced susceptibility was found to be associated with penicillin resistance. Higher antimicrobial susceptibility was seen with *H. influenzae* in both countries. Susceptibility has remained effectively unchanged since 2015–17 in *S. pneumoniae* isolates from Kuwait and since 2011–13 in *H. influenzae* isolates from the UAE. Continued surveillance of antibiotic susceptibility is required to regularly assess any future changes.

## Supplementary Material

dkaf284_Supplementary_Data

## Supplementary data

Tables S1–S4 are available as Supplementary data at *JAC* Online.
