# Supplementary material for: Results from the Survey of Antibiotic Resistance (SOAR) 2018–21 in Kuwait and the United Arab Emirates: data based on CLSI, EUCAST (dose-specific) and pharmacokinetic/pharmacodynamic (PK/PD) breakpoints
Source: J Antimicrob Chemother. 2025 Nov 24;80(Suppl 3):iii19–37. doi: 10.1093/jac/dkaf284 (PMC12641136; doi:10.1093/jac/dkaf284)
Supplement: dkaf284_Supplementary_Data [file dkaf284_supplementary_data.docx]

**Results from the Survey of Antibiotic Resistance (SOAR) 2018 – 21 in Kuwait and the United Arab Emirates: data based on CLSI, EUCAST (dose-specific) and pharmacokinetic/ pharmacodynamic (PK/PD) breakpoints**

**Authors:** Didem TORUMKUNEY^1^, Eiman MOKADDAS^2^, Stefan WEBER^3^, Ian MORRISSEY^4^, Nergis KELES^5^, Anand MANOHARAN^6^*

**Affiliations:** ^1^GSK, London, UK; ^2^Microbiology Department, Faculty of Medicine, Kuwait University, Jabrya, Kuwait; ^3^Microbiology Laboratory & Reference Laboratory for Infectious Diseases, Purelab Sheikh Khalifa Medical City, Abu Dhabi, United Arab Emirates; ^4^Antimicrobial Focus Ltd., Sawbridgeworth, UK; ^5^GSK, Levent Özdilek River Plaza No: 13 İç Kapı No: 61 Şişli, 34330 Beşiktaş/İstanbul, Türkiye; ^6^Infectious Diseases Medical & Scientific Affairs, GSK, Mumbai, India

*Corresponding author. E-mail: [anand.x.manoharan@gsk.com](mailto:anand.x.manoharan@gsk.com)

**Running title:** Survey of Antibiotic Resistance (SOAR) in Kuwait and UAE in 2018 – 21

**Supplementary Table 1.** MIC distribution data for *S. pneumoniae* isolates (*n* = 49) from Kuwait

|  |  | Number of isolates at MIC (mg/L) | | | | | | | | | | | | | | | | | | | | |
| --- | --- | --- | --- | --- | --- | --- | --- | --- | --- | --- | --- | --- | --- | --- | --- | --- | --- | --- | --- | --- | --- | --- |
| Antimicrobial | | ≤0.008 | ≤0.015 | 0.015 | ≤0.03 | 0.03 | ≤0.06 | 0.06 | ≤0.12 | 0.12 | ≤0.25 | 0.25 | ≤0.5 | 0.5 | 1 | 2 | 4 | >4 | 8 | >8 | 16 | >16 |
| AMX | N | – | – | 2 | – | 8 | – | – | – | 4 | – | 6 | – | 8 | 2 | 9 | 5 | – | 5 | – | – | – |
|  | Cum. % | – | – | 4.1 | – | 20.4 | – | – | – | 28.6 | – | 40.8 | – | 57.1 | 61.2 | 79.6 | 89.8 | – | 100 | – | – | – |
|  | % | – | – | 4.1 | – | 16.3 | – | – | – | 8.2 | – | 12.2 | – | 16.3 | 4.1 | 18.4 | 10.2 | – | 10.2 | – | – | – |
| AMC (2:1) | N | – | – | 4 | – | 6 | – | – | – | 4 | – | 7 | – | 7 | 3 | 9 | 4 | – | 5 | – | – | – |
|  | Cum. % | – | – | 8.2 | – | 20.4 | – | – | – | 28.6 | – | 42.9 | – | 57.1 | 63.3 | 81.6 | 89.8 | – | 100 | – | – | – |
|  | % | – | – | 8.2 | – | 12.2 | – | – | – | 8.2 | – | 14.3 | – | 14.3 | 6.1 | 18.4 | 8.2 | – | 10.2 | – | – | – |
| AMC  [2 mg/L] | N | – | – | – | – | 1 | – | 9 | – | – | – | 2 | – | 5 | 10 | 1 | 2 | – | 10 | 9 | – | – |
|  | Cum. % | – | – | – | – | 2.0 | – | 20.4 | – | – | – | 24.5 | – | 34.7 | 55.1 | 57.1 | 61.2 | – | 81.6 | 100 | – | – |
|  | % | – | – | – | – | 2.0 | – | 18.4 | – | – | – | 4.1 | – | 10.2 | 20.4 | 2.0 | 4.1 | – | 20.4 | 18.4 | – | – |
| AZM | N | – | – | – | – | – | – | – | – | 9 | – | 7 | – | – | – | – | 1 | – | – | – | 8 | 24 |
|  | Cum. % | – | – | – | – | – | – | – | – | 18.4 | – | 32.7 | – | – | – | – | 34.7 | – | – | – | 51.0 | 100 |
|  | % | – | – | – | – | – | – | – | – | 18.4 | – | 14.3 | – | – | – | – | 2.0 | – | – | – | 16.3 | 49.0 |
| CEC | N | – | – | – | – | – | – | – | – | – | – | – | – | 6 | 6 | 7 | 5 | 25 | – | – | – | – |
|  | Cum. % | – | – | – | – | – | – | – | – | – | – | – | – | 12.2 | 24.5 | 38.8 | 49.0 | 100 | – | – | – | – |
|  | % | – | – | – | – | – | – | – | – | – | – | – | – | 12.2 | 12.2 | 14.3 | 10.2 | 51.0 | – | – | – | – |
| CDR | N | – | – | – | – | – | – | 8 | – | 3 | – | 4 | – | 12 | 1 | – | 6 | – | 13 | 2 | – | – |
|  | Cum. % | – | – | – | – | – | – | 16.3 | – | 22.4 | – | 30.6 | – | 55.1 | 57.1 | – | 69.4 | – | 95.9 | 100 | – | – |
|  | % | – | – | – | – | – | – | 16.3 | – | 6.1 | – | 8.2 | – | 24.5 | 2.0 | – | 12.2 | – | 26.5 | 4.1 | – | – |
| CFM | N | – | – | – | – | – | – | – | – | – | 8 | – | – | 3 | 4 | 9 | 4 | – | – | – | 10 | 11 |
|  | Cum. % | – | – | – | – | – | – | – | – | – | 16.3 | – | – | 22.4 | 30.6 | 49.0 | 57.1 | – | – | – | 77.6 | 100 |
|  | % | – | – | – | – | – | – | – | – | – | 16.3 | – | – | 6.1 | 8.2 | 18.4 | 8.2 | – | – | – | 20.4 | 22.4 |
| CTX | N | – | – | 2 | – | 7 | – | 5 | – | 5 | – | 8 | – | 2 | 7 | 11 | 2 | – | – | – | – | – |
|  | Cum. % | – | – | 4.1 | – | 18.4 | – | 28.6 | – | 38.8 | – | 55.1 | – | 59.2 | 73.5 | 95.9 | 100 | – | – | – | – | – |
|  | % | – | – | 4.1 | – | 14.3 | – | 10.2 | – | 10.2 | – | 16.3 | – | 4.1 | 14.3 | 22.4 | 4.1 | – | – | – | – | – |
| CPD | N | – | – | – | – | 8 | – | 1 | – | 4 | – | 7 | – | 7 | 3 | 7 | 10 | 2 | – | – | – | – |
|  | Cum. % | – | – | – | – | 16.3 | – | 18.4 | – | 26.5 | – | 40.8 | – | 55.1 | 61.2 | 75.5 | 95.9 | 100 | – | – | – | – |
|  | % | – | – | – | – | 16.3 | – | 2.0 | – | 8.2 | – | 14.3 | – | 14.3 | 6.1 | 14.3 | 20.4 | 4.1 | – | – | – | – |
| CTB | N | – | – | – | – | – | – | – | – | – | – | – | – | – | – | – | 8 | – | 2 | – | 12 | 27 |
|  | Cum. % | – | – | – | – | – | – | – | – | – | – | – | – | – | – | – | 16.3 | – | 20.4 | – | 44.9 | 100 |
|  | % | – | – | – | – | – | – | – | – | – | – | – | – | – | – | – | 16.3 | – | 4.1 | – | 24.5 | 55.1 |
| CRO | N | – | – | 2 | – | 7 | – | 5 | – | 3 | – | 10 | – | 3 | 14 | 3 | 2 | – | – | – | – | – |
|  | Cum. % | – | – | 4.1 | – | 18.4 | – | 28.6 | – | 34.7 | – | 55.1 | – | 61.2 | 89.8 | 95.9 | 100 | – | – | – | – | – |
|  | % | – | – | 4.1 | – | 14.3 | – | 10.2 | – | 6.1 | – | 20.4 | – | 6.1 | 28.6 | 6.1 | 4.1 | – | – | – | – | – |
| CXM | N | – | – | 1 | – | 7 | – | 1 | – | – | – | 13 | – | 4 | 2 | – | 8 | – | 11 | 2 | – | – |
|  | Cum. % | – | – | 2.0 | – | 16.3 | – | 18.4 | – | – | – | 44.9 | – | 53.1 | 57.1 | – | 73.5 | – | 95.9 | 100 | – | – |
|  | % | – | – | 2.0 | – | 14.3 | – | 2.0 | – | – | – | 26.5 | – | 8.2 | 4.1 | – | 16.3 | – | 22.4 | 4.1 | – | – |
| CLR | N | – | – | – | – | 12 | – | 4 | – | – | – | – | – | 1 | – | – | 4 | – | 7 | – | 4 | 17 |
|  | Cum. % | – | – | – | – | 24.5 | – | 32.7 | – | – | – | – | – | 34.7 | – | – | 42.9 | – | 57.1 | – | 65.3 | 100 |
|  | % | – | – | – | – | 24.5 | – | 8.2 | – | – | – | – | – | 2.0 | – | – | 8.2 | – | 14.3 | – | 8.2 | 34.7 |
| DOX | N | – | – | – | – | – | – | 22 | – | 4 | – | – | – | – | – | 4 | 11 | 8 | – | – | – | – |
|  | Cum. % | – | – | – | – | – | – | 44.9 | – | 53.1 | – | – | – | – | – | 61.2 | 83.7 | 100 | – | – | – | – |
|  | % | – | – | – | – | – | – | 44.9 | – | 8.2 | – | – | – | – | – | 8.2 | 22.4 | 16.3 | – | – | – | – |
| ERY | N | – | – | – | – | 1 | – | 15 | – | – | – | – | – | – | – | 1 | 1 | – | 5 | – | 6 | 20 |
|  | Cum. % | – | – | – | – | 2.0 | – | 32.7 | – | – | – | – | – | – | – | 34.7 | 36.7 | – | 46.9 | – | 59.2 | 100 |
|  | % | – | – | – | – | 2.0 | – | 30.6 | – | – | – | – | – | – | – | 2.0 | 2.0 | – | 10.2 | – | 12.2 | 40.8 |
| LVX | N | – | – | – | – | – | – | – | – | – | – | – | – | 5 | 44 | – | – | – | – | – | – | – |
|  | Cum. % | – | – | – | – | – | – | – | – | – | – | – | – | 10.2 | 100 | – | – | – | – | – | – | – |
|  | % | – | – | – | – | – | – | – | – | – | – | – | – | 10.2 | 89.8 | – | – | – | – | – | – | – |
| MXF | N | – | – | – | – | – | – | 4 | – | 44 | – | 1 | – | – | – | – | – | – | – | – | – | – |
|  | Cum. % | – | – | – | – | – | – | 8.2 | – | 98.0 | – | 100 | – | – | – | – | – | – | – | – | – | – |
|  | % | – | – | – | – | – | – | 8.2 | – | 89.8 | – | 2.0 | – | – | – | – | – | – | – | – | – | – |
| PEN | N | – | – | 7 | – | 2 | – | 1 | – | 4 | – | 7 | – | 7 | 3 | 11 | 7 | – | – | – | – | – |
|  | Cum. % | – | – | 14.3 | – | 18.4 | – | 20.4 | – | 28.6 | – | 42.9 | – | 57.1 | 63.3 | 85.7 | 100 | – | – | – | – | – |
|  | % | – | – | 14.3 | – | 4.1 | – | 2.0 | – | 8.2 | – | 14.3 | – | 14.3 | 6.1 | 22.4 | 14.3 | – | – | – | – | – |
| TET | N | – | – | – | – | – | – | – | – | 20 | – | 6 | – | – | – | – | – | 23 | – | – | – | – |
|  | Cum. % | – | – | – | – | – | – | – | – | 40.8 | – | 53.1 | – | – | – | – | – | 100 | – | – | – | – |
|  | % | – | – | – | – | – | – | – | – | 40.8 | – | 12.2 | – | – | – | – | – | 46.9 | – | – | – | – |
| SXT | N | – | – | – | – | – | – | – | – | 1 | – | 14 | – | 1 | 9 | 4 | 2 | – | 14 | 4 | – | – |
|  | Cum. % | – | – | – | – | – | – | – | – | 2.0 | – | 30.6 | – | 32.7 | 51.0 | 59.2 | 63.3 | – | 91.8 | 100 | – | – |
|  | % | – | – | – | – | – | – | – | – | 2.0 | – | 28.6 | – | 2.0 | 18.4 | 8.2 | 4.1 | – | 28.6 | 8.2 | – | – |

–, not applicable; AMC, amoxicillin/clavulanic acid; AMX, amoxicillin; AZM, azithromycin; CDR, cefdinir; CEC, cefaclor; CFM, cefixime; CLR, clarithromycin; CPD, cefpodoxime; CRO, ceftriaxone; CTB, ceftibuten; CTX, cefotaxime; Cum., cumulative; CXM, cefuroxime; DOX, doxycycline; ERY, erythromycin; LVX, levofloxacin; MXF, moxifloxacin; PEN, penicillin; SXT, trimethoprim/sulfamethoxazole; TET, tetracycline.

Bold vertical bars in table correspond to the CLSI-susceptible breakpoints.

**Supplementary Table 2.** MIC distribution data for *S. pneumoniae* isolates (*n* = 49) from the UAE

|  |  | Number of isolates at MIC (mg/L) | | | | | | | | | | | | | | | | | | | | |
| --- | --- | --- | --- | --- | --- | --- | --- | --- | --- | --- | --- | --- | --- | --- | --- | --- | --- | --- | --- | --- | --- | --- |
| Antimicrobial | | ≤0.008 | ≤0.015 | 0.015 | ≤0.03 | 0.03 | ≤0.06 | 0.06 | ≤0.12 | 0.12 | ≤0.25 | 0.25 | ≤0.5 | 0.5 | 1 | 2 | 4 | >4 | 8 | >8 | 16 | >16 |
| AMX | N | 1 | – | 5 | – | 5 | – | 1 | – | 7 | – | 13 | – | 3 | 6 | 4 | 4 | – | – | – | – | – |
|  | Cum. % | 2.0 | – | 12.2 | – | 22.4 | – | 24.5 | – | 38.8 | – | 65.3 | – | 71.4 | 83.7 | 91.8 | 100 | – | – | – | – | – |
|  | % | 2.0 | – | 10.2 | – | 10.2 | – | 2.0 | – | 14.3 | – | 26.5 | – | 6.1 | 12.2 | 8.2 | 8.2 | – | – | – | – | – |
| AMC (2:1) | N | 2 | – | 5 | – | 4 | – | 1 | – | 7 | – | 13 | – | 3 | 6 | 5 | 3 | – | – | – | – | – |
|  | Cum. % | 4.1 | – | 14.3 | – | 22.4 | – | 24.5 | – | 38.8 | – | 65.3 | – | 71.4 | 83.7 | 93.9 | 100 | – | – | – | – | – |
|  | % | 4.1 | – | 10.2 | – | 8.2 | – | 2.0 | – | 14.3 | – | 26.5 | – | 6.1 | 12.2 | 10.2 | 6.1 | – | – | – | – | – |
| AMC  [2 mg/L] | N | 1 | – | 1 | – | 4 | – | 5 | – | 1 | – | 7 | – | 5 | 12 | 1 | 5 | – | 5 | 2 | – | – |
|  | Cum. % | 2.0 | – | 4.1 | – | 12.2 | – | 22.4 | – | 24.5 | – | 38.8 | – | 49.0 | 73.5 | 75.5 | 85.7 | – | 95.9 | 100 | – | – |
|  | % | 2.0 | – | 2.0 | – | 8.2 | – | 10.2 | – | 2.0 | – | 14.3 | – | 10.2 | 24.5 | 2.0 | 10.2 | – | 10.2 | 4.1 | – | – |
| AZM | N | – | 2 | – | – | 7 | – | 12 | – | 2 | – | – | – | 1 | 2 | 5 | 2 | – | 2 | – | 2 | 12 |
|  | Cum. % | – | 4.1 | – | – | 18.4 | – | 42.9 | – | 46.9 | – | – | – | 49.0 | 53.1 | 63.3 | 67.3 | – | 71.4 | – | 75.5 | 100 |
|  | % | – | 4.1 | – | – | 14.3 | – | 24.5 | – | 4.1 | – | – | – | 2.0 | 4.1 | 10.2 | 4.1 | – | 4.1 | – | 4.1 | 24.5 |
| CEC | N | – | – | – | – | – | – | – | – | 1 | – | 1 | – | 6 | 9 | 4 | 12 | 16 | – | – | – | – |
|  | Cum. % | – | – | – | – | – | – | – | – | 2.0 | – | 4.1 | – | 16.3 | 34.7 | 42.9 | 67.3 | 100 | – | – | – | – |
|  | % | – | – | – | – | – | – | – | – | 2.0 | – | 2.0 | – | 12.2 | 18.4 | 8.2 | 24.5 | 32.7 | – | – | – | – |
| CDR | N | – | – | – | – | 3 | – | 8 | – | 2 | – | 9 | – | 8 | 4 | 2 | 6 | – | 5 | 2 | – | – |
|  | Cum. % | – | – | – | – | 6.1 | – | 22.4 | – | 26.5 | – | 44.9 | – | 61.2 | 69.4 | 73.5 | 85.7 | – | 95.9 | 100 | – | – |
|  | % | – | – | – | – | 6.1 | – | 16.3 | – | 4.1 | – | 18.4 | – | 16.3 | 8.2 | 4.1 | 12.2 | – | 10.2 | 4.1 | – | – |
| CFM | N | – | – | – | – | – | – | – | – | – | 10 | – | – | 5 | 6 | 9 | 5 | – | 4 | – | 6 | 4 |
|  | Cum. % | – | – | – | – | – | – | – | – | – | 20.4 | – | – | 30.6 | 42.9 | 61.2 | 71.4 | – | 79.6 | – | 91.8 | 100 |
|  | % | – | – | – | – | – | – | – | – | – | 20.4 | – | – | 10.2 | 12.2 | 18.4 | 10.2 | – | 8.2 | – | 12.2 | 8.2 |
| CTX | N | 1 | – | 7 | – | 2 | – | 6 | – | 8 | – | 8 | – | 6 | 5 | 4 | 2 | – | – | – | – | – |
|  | Cum. % | 2.0 | – | 16.3 | – | 20.4 | – | 32.7 | – | 49.0 | – | 65.3 | – | 77.6 | 87.8 | 95.9 | 100 | – | – | – | – | – |
|  | % | 2.0 | – | 14.3 | – | 4.1 | – | 12.2 | – | 16.3 | – | 16.3 | – | 12.2 | 10.2 | 8.2 | 4.1 | – | – | – | – | – |
| CPD | N | – | 3 | – | – | 7 | – | 3 | – | 6 | – | 9 | – | 6 | 4 | 5 | 4 | 2 | – | – | – | – |
|  | Cum. % | – | 6.1 | – | – | 20.4 | – | 26.5 | – | 38.8 | – | 57.1 | – | 69.4 | 77.6 | 87.8 | 95.9 | 100 | – | – | – | – |
|  | % | – | 6.1 | – | – | 14.3 | – | 6.1 | – | 12.2 | – | 18.4 | – | 12.2 | 8.2 | 10.2 | 8.2 | 4.1 | – | – | – | – |
| CTB | N | – | – | – | – | – | – | – | – | – | – | – | – | – | – | 2 | 9 | – | 8 | – | 9 | 21 |
|  | Cum. % | – | – | – | – | – | – | – | – | – | – | – | – | – | – | 4.1 | 22.4 | – | 38.8 | – | 57.1 | 100 |
|  | % | – | – | – | – | – | – | – | – | – | – | – | – | – | – | 4.1 | 18.4 | – | 16.3 | – | 18.4 | 42.9 |
| CRO | N | – | – | 6 | – | 4 | – | 3 | – | 7 | – | 10 | – | 7 | 9 | 2 | 1 | – | – | – | – | – |
|  | Cum. % | – | – | 12.2 | – | 20.4 | – | 26.5 | – | 40.8 | – | 61.2 | – | 75.5 | 93.9 | 98.0 | 100 | – | – | – | – | – |
|  | % | – | – | 12.2 | – | 8.2 | – | 6.1 | – | 14.3 | – | 20.4 | – | 14.3 | 18.4 | 4.1 | 2.0 | – | – | – | – | – |
| CXM | N | – | – | 4 | – | 5 | – | 2 | – | 6 | – | 10 | – | 5 | 2 | 4 | 6 | – | 3 | 2 | – | – |
|  | Cum. % | – | – | 8.2 | – | 18.4 | – | 22.4 | – | 34.7 | – | 55.1 | – | 65.3 | 69.4 | 77.6 | 89.8 | – | 95.9 | 100 | – | – |
|  | % | – | – | 8.2 | – | 10.2 | – | 4.1 | – | 12.2 | – | 20.4 | – | 10.2 | 4.1 | 8.2 | 12.2 | – | 6.1 | 4.1 | – | – |
| CLR | N | – | 14 | – | – | 9 | – | – | – | – | – | 1 | – | 1 | 6 | 1 | 2 | – | 3 | – | 1 | 11 |
|  | Cum. % | – | 28.6 | – | – | 46.9 | – | – | – | – | – | 49.0 | – | 51.0 | 63.3 | 65.3 | 69.4 | – | 75.5 | – | 77.6 | 100 |
|  | % | – | 28.6 | – | – | 18.4 | – | – | – | – | – | 2.0 | – | 2.0 | 12.2 | 2.0 | 4.1 | – | 6.1 | – | 2.0 | 22.4 |
| DOX | N | – | – | – | – | 1 | – | 8 | – | 19 | – | – | – | 1 | – | 1 | 7 | 12 | – | – | – | – |
|  | Cum. % | – | – | – | – | 2.0 | – | 18.4 | – | 57.1 | – | – | – | 59.2 | – | 61.2 | 75.5 | 100 | – | – | – | – |
|  | % | – | – | – | – | 2.0 | – | 16.3 | – | 38.8 | – | – | – | 2.0 | – | 2.0 | 14.3 | 24.5 | – | – | – | – |
| ERY | N | – | 6 | – | – | 15 | – | 2 | – | – | – | – | – | 1 | – | 7 | 2 | – | 1 | – | 3 | 12 |
|  | Cum. % | – | 12.2 | – | – | 42.9 | – | 46.9 | – | – | – | – | – | 49.0 | – | 63.3 | 67.3 | – | 69.4 | – | 75.5 | 100 |
|  | % | – | 12.2 | – | – | 30.6 | – | 4.1 | – | – | – | – | – | 2.0 | – | 14.3 | 4.1 | – | 2.0 | – | 6.1 | 24.5 |
| LVX | N | – | – | – | – | – | – | – | – | – | – | – | – | 7 | 38 | 3 | – | – | – | 1 | – | – |
|  | Cum. % | – | – | – | – | – | – | – | – | – | – | – | – | 14.3 | 91.8 | 98.0 | – | – | – | 100 | – | – |
|  | % | – | – | – | – | – | – | – | – | – | – | – | – | 14.3 | 77.6 | 6.1 | – | – | – | 2.0 | – | – |
| MXF | N | – | – | – | 1 | – | – | 2 | – | 39 | – | 6 | – | – | 1 | – | – | – | – | – | – | – |
|  | Cum. % | – | – | – | 2.0 | – | – | 6.1 | – | 85.7 | – | 98.0 | – | – | 100 | – | – | – | – | – | – | – |
|  | % | – | – | – | 2.0 | – | – | 4.1 | – | 79.6 | – | 12.2 | – | – | 2.0 | – | – | – | – | – | – | – |
| PEN | N | 2 | – | 6 | – | 3 | – | 2 | – | 7 | – | 10 | – | 5 | 6 | 6 | 1 | – | 1 | – | – | – |
|  | Cum. % | 4.1 | – | 16.3 | – | 22.4 | – | 26.5 | – | 40.8 | – | 61.2 | – | 71.4 | 83.7 | 95.9 | 98.0 | – | 100 | – | – | – |
|  | % | 4.1 | – | 12.2 | – | 6.1 | – | 4.1 | – | 14.3 | – | 20.4 | – | 10.2 | 12.2 | 12.2 | 2.0 | – | 2.0 | – | – | – |
| TET | N | – | – | – | – | – | – | – | – | 6 | – | 19 | – | 2 | – | – | 1 | 21 | – | – | – | – |
|  | Cum. % | – | – | – | – | – | – | – | – | 12.2 | – | 51.0 | – | 55.1 | – | – | 57.1 | 100 | – | – | – | – |
|  | % | – | – | – | – | – | – | – | – | 12.2 | – | 38.8 | – | 4.1 | – | – | 2.0 | 42.9 | – | – | – | – |
| SXT | N | – | – | – | – | – | 2 | – | – | 6 | – | 13 | – | 2 | 5 | 2 | 7 | – | 12 | – | – | – |
|  | Cum. % | – | – | – | – | – | 4.1 | – | – | 16.3 | – | 42.9 | – | 46.9 | 57.1 | 61.2 | 75.5 | – | 100 | – | – | – |
|  | % | – | – | – | – | – | 4.1 | – | – | 12.2 | – | 26.5 | – | 4.1 | 10.2 | 4.1 | 14.3 | – | 24.5 | – | – | – |

–, not applicable; AMC, amoxicillin/clavulanic acid; AMX, amoxicillin; AZM, azithromycin; CDR, cefdinir; CEC, cefaclor; CFM, cefixime; CLR, clarithromycin; CPD, cefpodoxime; CRO, ceftriaxone; CTB, ceftibuten; CTX, cefotaxime; Cum., cumulative; CXM, cefuroxime; DOX, doxycycline; ERY, erythromycin; LVX, levofloxacin; MXF, moxifloxacin; PEN, penicillin; SXT, trimethoprim/sulfamethoxazole; TET, tetracycline.

Bold vertical bars in table correspond to the CLSI-susceptible breakpoints.

**Supplementary Table 3.** MIC distribution data for *H. influenzae* isolates (*n* = 79) from Kuwait

|  |  | |  | | Number of isolates at MIC (mg/L) | | | | | | | | | | | | | | | | | | | | | | | | | | | | |
| --- | --- | --- | --- | --- | --- | --- | --- | --- | --- | --- | --- | --- | --- | --- | --- | --- | --- | --- | --- | --- | --- | --- | --- | --- | --- | --- | --- | --- | --- | --- | --- | --- | --- |
| Antimicrobial | | ≤0.001 | ≤0.002 | 0.002 | | ≤0.004 | 0.004 | ≤0.008 | 0.008 | ≤0.015 | 0.015 | ≤0.03 | 0.03 | ≤0.06 | 0.06 | ≤0.12 | 0.12 | ≤0.25 | 0.25 | 0.5 | 1 | 2 | 4 | >4 | 8 | >8 | 16 | >16 | 32 | >32 | 64 | 128 | >128 |
| AMX | N | – | – | – | | – | – | – | – | – | – | 1 | – | – | 2 | – | 2 | – | 15 | 21 | 13 | 7 | 4 | – | – | – | 3 | – | 2 | – | 3 | 3 | 3 |
|  | Cum. % | – | – | – | | – | – | – | – | – | – | 1.3 | – | – | 3.8 | – | 6.3 | – | 25.3 | 51.9 | 68.4 | 77.2 | 82.3 | – | – | – | 86.1 | – | 88.6 | – | 92.4 | 96.2 | 100 |
|  | % | – | – | – | | – | – | – | – | – | – | 1.3 | – | – | 2.5 | – | 2.5 | – | 19.0 | 26.6 | 16.5 | 8.9 | 5.1 | – | – | – | 3.8 | – | 2.5 | – | 3.8 | 3.8 | 3.8 |
| AMC (2:1) | N | – | – | – | | – | – | – | – | – | – | – | – | – | – | – | 3 | – | 5 | 22 | 18 | 16 | 13 | – | – | – | 1 | – | 1 | – | – | – | – |
|  | Cum. % | – | – | – | | – | – | – | – | – | – | – | – | – | – | – | 3.8 | – | 10.1 | 38.0 | 60.8 | 81.0 | 97.5 | – | – | – | 98.7 | – | 100 | – | – | – | – |
|  | % | – | – | – | | – | – | – | – | – | – | – | – | – | – | – | 3.8 | – | 6.3 | 27.8 | 22.8 | 20.3 | 16.5 | – | – | – | 1.3 | – | 1.3 | – | – | – | – |
| AMC  [2 mg/L] | N | – | – | – | | – | – | – | – | – | – | 3 | – | – | 2 | – | 4 | – | 18 | 23 | 14 | 10 | 3 | – | – | – | 2 | – | – | – | – | – | – |
|  | Cum. % | – | – | – | | – | – | – | – | – | – | 3.8 | – | – | 6.3 | – | 11.4 | – | 34.2 | 63.3 | 81.0 | 93.7 | 97.5 | – | – | – | 100 | – | – | – | – | – | – |
|  | % | – | – | – | | – | – | – | – | – | – | 3.8 | – | – | 2.5 | – | 5.1 | – | 22.8 | 29.1 | 17.7 | 12.7 | 3.8 | – | – | – | 2.5 | – | – | – | – | – | – |
| AMP | N | – | – | – | | – | – | – | – | – | – | 3 | – | – | – | – | 14 | – | 21 | 7 | 19 | 1 | – | – | 2 | – | 1 | – | 1 | – | 3 | 4 | 3 |
|  | Cum. % | – | – | – | | – | – | – | – | – | – | 3.8 | – | – | – | – | 21.5 | – | 48.1 | 57.0 | 81.0 | 82.3 | – | – | 84.8 | – | 86.1 | – | 87.3 | – | 91.1 | 96.2 | 100 |
|  | % | – | – | – | | – | – | – | – | – | – | 3.8 | – | – | – | – | 17.7 | – | 26.6 | 8.9 | 24.1 | 1.3 | – | – | 2.5 | – | 1.3 | – | 1.3 | – | 3.8 | 5.1 | 3.8 |
| AZM | N | – | – | – | | – | – | – | – | – | – | – | – | – | – | 4 | – | – | 2 | 12 | 40 | 18 | – | – | – | 3 | – | – | – | – | – | – | – |
|  | Cum. % | – | – | – | | – | – | – | – | – | – | – | – | – | – | 5.1 | – | – | 7.6 | 22.8 | 73.4 | 96.2 | – | – | – | 100 | – | – | – | – | – | – | – |
|  | % | – | – | – | | – | – | – | – | – | – | – | – | – | – | 5.1 | – | – | 2.5 | 15.2 | 50.6 | 22.8 | – | – | – | 3.8 | – | – | – | – | – | – | – |
| CEC | N | – | – | – | | – | – | – | – | – | – | – | – | – | – | – | – | 1 | – | 6 | 12 | 27 | 17 | – | 7 | – | 6 | – | – | 3 | – | – | – |
|  | Cum. % | – | – | – | | – | – | – | – | – | – | – | – | – | – | – | – | 1.3 | – | 8.9 | 24.1 | 58.2 | 79.7 | – | 88.6 | – | 96.2 | – | – | 100 | – | – | – |
|  | % | – | – | – | | – | – | – | – | – | – | – | – | – | – | – | – | 1.3 | – | 7.6 | 15.2 | 34.2 | 21.5 | – | 8.9 | – | 7.6 | – | – | 3.8 | – | – | – |
| CDR | N | – | – | – | | – | – | – | – | – | – | – | – | 10 | – | – | 15 | – | 38 | 11 | 2 | – | 1 | 2 | – | – | – | – | – | – | – | – | – |
|  | Cum. % | – | – | – | | – | – | – | – | – | – | – | – | 12.7 | – | – | 31.6 | – | 79.7 | 93.7 | 96.2 | – | 97.5 | 100 | – | – | – | – | – | – | – | – | – |
|  | % | – | – | – | | – | – | – | – | – | – | – | – | 12.7 | – | – | 19.0 | – | 48.1 | 13.9 | 2.5 | – | 1.3 | 2.5 | – | – | – | – | – | – | – | – | – |
| CFM | N | – | – | – | | – | – | 9 | – | – | 23 | – | 38 | – | 4 | – | 2 | – | – | 1 | – | 2 | – | – | – | – | – | – | – | – | – | – | – |
|  | Cum. % | – | – | – | | – | – | 11.4 | – | – | 40.5 | – | 88.6 | – | 93.7 | – | 96.2 | – | – | 97.5 | – | 100 | – | – | – | – | – | – | – | – | – | – | – |
|  | % | – | – | – | | – | – | 11.4 | – | – | 29.1 | – | 48.1 | – | 5.1 | – | 2.5 | – | – | 1.3 | – | 2.5 | – | – | – | – | – | – | – | – | – | – | – |
| CTX | N | – | 27 | – | | – | 25 | – | 10 | – | 11 | – | 2 | – | 2 | – | 2 | – | – | – | – | – | – | – | – | – | – | – | – | – | – | – | – |
|  | Cum. % | – | 34.2 | – | | – | 65.8 | – | 78.5 | – | 92.4 | – | 94.9 | – | 97.5 | – | 100 | – | – | – | – | – | – | – | – | – | – | – | – | – | – | – | – |
|  | % | – | 34.2 | – | | – | 31.6 | – | 12.7 | – | 13.9 | – | 2.5 | – | 2.5 | – | 2.5 | – | – | – | – | – | – | – | – | – | – | – | – | – | – | – | – |
| CPD | N | – | – | – | | – | – | – | – | 8 | – | – | 17 | – | 30 | – | 15 | – | 3 | 1 | 2 | 2 | 1 | – | – | – | – | – | – | – | – | – | – |
|  | Cum. % | – | – | – | | – | – | – | – | 10.1 | – | – | 31.6 | – | 69.6 | – | 88.6 | – | 92.4 | 93.7 | 96.2 | 98.7 | 100 | – | – | – | – | – | – | – | – | – | – |
|  | % | – | – | – | | – | – | – | – | 10.1 | – | – | 21.5 | – | 38.0 | – | 19.0 | – | 3.8 | 1.3 | 2.5 | 2.5 | 1.3 | – | – | – | – | – | – | – | – | – | – |
| CTB | N | – | – | – | | – | – | 4 | – | – | 4 | – | 26 | – | 23 | – | 12 | – | 5 | – | 1 | 1 | – | 3 | – | – | – | – | – | – | – | – | – |
|  | Cum. % | – | – | – | | – | – | 5.1 | – | – | 10.1 | – | 43.0 | – | 72.2 | – | 87.3 | – | 93.7 | – | 94.9 | 96.2 | – | 100 | – | – | – | – | – | – | – | – | – |
|  | % | – | – | – | | – | – | 5.1 | – | – | 5.1 | – | 32.9 | – | 29.1 | – | 15.2 | – | 6.3 | – | 1.3 | 1.3 | – | 3.8 | – | – | – | – | – | – | – | – | – |
| CRO | N | 12 | – | 17 | | – | 26 | – | 16 | – | 3 | – | 2 | – | 2 | – | 1 | – | – | – | – | – | – | – | – | – | – | – | – | – | – | – | – |
|  | Cum. % | 15.2 | – | 36.7 | | – | 69.6 | – | 89.9 | – | 93.7 | – | 96.2 | – | 98.7 | – | 100 | – | – | – | – | – | – | – | – | – | – | – | – | – | – | – | – |
|  | % | 15.2 | – | 21.5 | | – | 32.9 | – | 20.3 | – | 3.8 | – | 2.5 | – | 2.5 | – | 1.3 | – | – | – | – | – | – | – | – | – | – | – | – | – | – | – | – |
| CXM | N | – | – | – | | – | – | – | – | – | – | 2 | – | – | 3 | – | 4 | – | 13 | 28 | 12 | 12 | 2 | – | – | – | 2 | 1 | – | – | – | – | – |
|  | Cum. % | – | – | – | | – | – | – | – | – | – | 2.5 | – | – | 6.3 | – | 11.4 | – | 27.8 | 63.3 | 78.5 | 93.7 | 96.2 | – | – | – | 98.7 | 100 | – | – | – | – | – |
|  | % | – | – | – | | – | – | – | – | – | – | 2.5 | – | – | 3.8 | – | 5.1 | – | 16.5 | 35.4 | 15.2 | 15.2 | 2.5 | – | – | – | 2.5 | 1.3 | – | – | – | – | – |
| CLR | N | – | – | – | | – | – | – | – | – | – | – | – | – | – | – | – | 1 | – | 2 | 1 | 7 | 44 | – | 20 | – | 1 | – | – | 3 | – | – | – |
|  | Cum. % | – | – | – | | – | – | – | – | – | – | – | – | – | – | – | – | 1.3 | – | 3.8 | 5.1 | 13.9 | 69.6 | – | 94.9 | – | 96.2 | – | – | 100 | – | – | – |
|  | % | – | – | – | | – | – | – | – | – | – | – | – | – | – | – | – | 1.3 | – | 2.5 | 1.3 | 8.9 | 55.7 | – | 25.3 | – | 1.3 | – | – | 3.8 | – | – | – |
| LVX | N | – | – | – | | 6 | – | – | 6 | – | 50 | – | 4 | – | – | – | 1 | – | 1 | 7 | 3 | – | – | – | 1 | – | – | – | – | – | – | – | – |
|  | Cum. % | – | – | – | | 7.6 | – | – | 15.2 | – | 78.5 | – | 83.5 | – | – | – | 84.8 | – | 86.1 | 94.9 | 98.7 | – | – | – | 100 | – | – | – | – | – | – | – | – |
|  | % | – | – | – | | 7.6 | – | – | 7.6 | – | 63.3 | – | 5.1 | – | – | – | 1.3 | – | 1.3 | 8.9 | 3.8 | – | – | – | 1.3 | – | – | – | – | – | – | – | – |
| MXF | N | – | – | – | | 6 | – | – | 14 | – | 28 | – | 18 | – | – | – | 1 | – | 2 | 4 | 5 | – | – | – | 1 | – | – | – | – | – | – | – | – |
|  | Cum. % | – | – | – | | 7.6 | – | – | 25.3 | – | 60.8 | – | 83.5 | – | – | – | 84.8 | – | 87.3 | 92.4 | 98.7 | – | – | – | 100 | – | – | – | – | – | – | – | – |
|  | % | – | – | – | | 7.6 | – | – | 17.7 | – | 35.4 | – | 22.8 | – | – | – | 1.3 | – | 2.5 | 5.1 | 6.3 | – | – | – | 1.3 | – | – | – | – | – | – | – | – |
| TET | N | – | – | – | | – | – | – | – | – | – | – | – | – | – | 5 | – | – | 71 | 1 | – | – | 1 | – | – | – | – | – | 1 | – | – | – | – |
|  | Cum. % | – | – | – | | – | – | – | – | – | – | – | – | – | – | 6.3 | – | – | 96.2 | 97.5 | – | – | 98.7 | – | – | – | – | – | 100 | – | – | – | – |
|  | % | – | – | – | | – | – | – | – | – | – | – | – | – | – | 6.3 | – | – | 89.9 | 1.3 | – | – | 1.3 | – | – | – | – | – | 1.3 | – | – | – | – |
| SXT | N | – | – | – | | – | – | 2 | – | – | 1 | – | 2 | – | 15 | – | 20 | – | 3 | 1 | 1 | 3 | 5 | – | 14 | 12 | – | – | – | – | – | – | – |
|  | Cum. % | – | – | – | | – | – | 2.5 | – | – | 3.8 | – | 6.3 | – | 25.3 | – | 50.6 | – | 54.4 | 55.7 | 57.0 | 60.8 | 67.1 | – | 84.8 | 100 | – | – | – | – | – | – | – |
|  | % | – | – | – | | – | – | 2.5 | – | – | 1.3 | – | 2.5 | – | 19.0 | – | 25.3 | – | 3.8 | 1.3 | 1.3 | 3.8 | 6.3 | – | 17.7 | 15.2 | – | – | – | – | – | – | – |

–, not applicable; AMC, amoxicillin/clavulanic acid; AMP, ampicillin; AMX, amoxicillin; AZM, azithromycin; CDR, cefdinir; CEC, cefaclor; CFM, cefixime; CLR, clarithromycin; CPD, cefpodoxime; CRO, ceftriaxone; CTB, ceftibuten; CTX, cefotaxime; Cum., cumulative; CXM, cefuroxime; LVX, levofloxacin; MXF, moxifloxacin; SXT, trimethoprim/sulfamethoxazole; TET, tetracycline.

Bold vertical bars in table correspond to the CLSI-susceptible breakpoints.

**Supplementary Table 4.** MIC distribution data for *H. influenzae* isolates (*n* = 34) from the UAE

|  |  | Number of isolates at MIC (mg/L) | | | | | | | | | | | | | | | | | | | | | | | | | | | | | |
| --- | --- | --- | --- | --- | --- | --- | --- | --- | --- | --- | --- | --- | --- | --- | --- | --- | --- | --- | --- | --- | --- | --- | --- | --- | --- | --- | --- | --- | --- | --- | --- |
| Antimicrobial | | ≤0.001 | ≤0.002 | 0.002 | ≤0.004 | 0.004 | ≤0.008 | 0.008 | ≤0.015 | 0.015 | ≤0.03 | 0.03 | ≤0.06 | 0.06 | ≤0.12 | 0.12 | ≤0.25 | 0.25 | 0.5 | 1 | 2 | 4 | >4 | 8 | >8 | 16 | 32 | >32 | 64 | 128 | >128 |
| AMX | N | – | – | – | – | – | – | – | – | – | – | – | – | – | – | 4 | – | 8 | 13 | 4 | 2 | – | – | 1 | – | – | – | – | 2 | – | – |
|  | Cum. % | – | – | – | – | – | – | – | – | – | – | – | – | – | – | 11.8 | – | 35.3 | 73.5 | 85.3 | 91.2 | – | – | 94.1 | – | – | – | – | 100 | – | – |
|  | % | – | – | – | – | – | – | – | – | – | – | – | – | – | – | 11.8 | – | 23.5 | 38.2 | 11.8 | 5.9 | – | – | 2.9 | – | – | – | – | 5.9 | – | – |
| AMC (2:1) | N | – | – | – | – | – | – | – | – | – | – | – | – | – | – | 2 | – | 6 | 17 | 6 | 2 | 1 | – | – | – | – | – | – | – | – | – |
|  | Cum. % | – | – | – | – | – | – | – | – | – | – | – | – | – | – | 5.9 | – | 23.5 | 73.5 | 91.2 | 97.1 | 100 | – | – | – | – | – | – | – | – | – |
|  | % | – | – | – | – | – | – | – | – | – | – | – | – | – | – | 5.9 | – | 17.6 | 50.0 | 17.6 | 5.9 | 2.9 | – | – | – | – | – | – | – | – | – |
| AMC  [2 mg/L] | N | – | – | – | – | – | – | – | – | – | – | – | – | 1 | – | 3 | – | 16 | 9 | 3 | 2 | – | – | – | – | – | – | – | – | – | – |
|  | Cum. % | – | – | – | – | – | – | – | – | – | – | – | – | 2.9 | – | 11.8 | – | 58.8 | 85.3 | 94.1 | 100 | – | – | – | – | – | – | – | – | – | – |
|  | % | – | – | – | – | – | – | – | – | – | – | – | – | 2.9 | – | 8.8 | – | 47.1 | 26.5 | 8.8 | 5.9 | – | – | – | – | – | – | – | – | – | – |
| AMP | N | – | – | – | – | – | – | – | – | – | – | – | – | 1 | – | 10 | – | 12 | 4 | 3 | 1 | – | – | 1 | – | – | 1 | – | 1 | – | – |
|  | Cum. % | – | – | – | – | – | – | – | – | – | – | – | – | 2.9 | – | 32.4 | – | 67.6 | 79.4 | 88.2 | 91.2 | – | – | 94.1 | – | – | 97.1 | – | 100 | – | – |
|  | % | – | – | – | – | – | – | – | – | – | – | – | – | 2.9 | – | 29.4 | – | 35.3 | 11.8 | 8.8 | 2.9 | – | – | 2.9 | – | – | 2.9 | – | 2.9 | – | – |
| AZM | N | – | – | – | – | – | – | – | – | – | – | – | – | – | 3 | – | – | – | 9 | 13 | 7 | – | – | – | 2 | – | – | – | – | – | – |
|  | Cum. % | – | – | – | – | – | – | – | – | – | – | – | – | – | 8.8 | – | – | – | 35.3 | 73.5 | 94.1 | – | – | – | 100 | – | – | – | – | – | – |
|  | % | – | – | – | – | – | – | – | – | – | – | – | – | – | 8.8 | – | – | – | 26.5 | 38.2 | 20.6 | – | – | – | 5.9 | – | – | – | – | – | – |
| CEC | N | – | – | – | – | – | – | – | – | – | – | – | – | – | – | – | – | – | 3 | 12 | 9 | 7 | – | 1 | – | 1 | 1 | – | – | – | – |
|  | Cum. % | – | – | – | – | – | – | – | – | – | – | – | – | – | – | – | – | – | 8.8 | 44.1 | 70.6 | 91.2 | – | 94.1 | – | 97.1 | 100 | – | – | – | – |
|  | % | – | – | – | – | – | – | – | – | – | – | – | – | – | – | – | – | – | 8.8 | 35.3 | 26.5 | 20.6 | – | 2.9 | – | 2.9 | 2.9 | – | – | – | – |
| CDN | N | – | – | – | – | – | – | – | – | – | – | – | 3 | – | – | 9 | – | 13 | 7 | 2 | – | – | – | – | – | – | – | – | – | – | – |
|  | Cum. % | – | – | – | – | – | – | – | – | – | – | – | 8.8 | – | – | 35.3 | – | 73.5 | 94.1 | 100 | – | – | – | – | – | – | – | – | – | – | – |
|  | % | – | – | – | – | – | – | – | – | – | – | – | 8.8 | – | – | 26.5 | – | 38.2 | 20.6 | 5.9 | – | – | – | – | – | – | – | – | – | – | – |
| CFM | N | – | – | – | – | – | 3 | – | – | 10 | – | 12 | – | 7 | – | 1 | – | 1 | – | – | – | – | – | – | – | – | – | – | – | – | – |
|  | Cum. % | – | – | – | – | – | 8.8 | – | – | 38.2 | – | 73.5 | – | 94.1 | – | 97.1 | – | 100 | – | – | – | – | – | – | – | – | – | – | – | – | – |
|  | % | – | – | – | – | – | 8.8 | – | – | 29.4 | – | 35.3 | – | 20.6 | – | 2.9 | – | 2.9 | – | – | – | – | – | – | – | – | – | – | – | – | – |
| CTX | N | – | 6 | – | – | 3 | – | 8 | – | 13 | – | 3 | – | 1 | – | – | – | – | – | – | – | – | – | – | – | – | – | – | – | – | – |
|  | Cum. % | – | 17.6 | – | – | 26.5 | – | 50.0 | – | 88.2 | – | 97.1 | – | 100 | – | – | – | – | – | – | – | – | – | – | – | – | – | – | – | – | – |
|  | % | – | 17.6 | – | – | 8.8 | – | 23.5 | – | 38.2 | – | 8.8 | – | 2.9 | – | – | – | – | – | – | – | – | – | – | – | – | – | – | – | – | – |
| CPD | N | – | – | – | – | – | – | – | 2 | – | – | 13 | – | 11 | – | 4 | – | 4 | – | – | – | – | – | – | – | – | – | – | – | – | – |
|  | Cum. % | – | – | – | – | – | – | – | 5.9 | – | – | 44.1 | – | 76.5 | – | 88.2 | – | 100 | – | – | – | – | – | – | – | – | – | – | – | – | – |
|  | % | – | – | – | – | – | – | – | 5.9 | – | – | 38.2 | – | 32.4 | – | 11.8 | – | 11.8 | – | – | – | – | – | – | – | – | – | – | – | – | – |
| CTB | N | – | – | – | – | – | 2 | – | – | – | – | 9 | – | 10 | – | 7 | – | 3 | 2 | 1 | – | – | – | – | – | – | – | – | – | – | – |
|  | Cum. % | – | – | – | – | – | 5.9 | – | – | – | – | 32.4 | – | 61.8 | – | 82.4 | – | 91.2 | 97.1 | 100 | – | – | – | – | – | – | – | – | – | – | – |
|  | % | – | – | – | – | – | 5.9 | – | – | – | – | 26.5 | – | 29.4 | – | 20.6 | – | 8.8 | 5.9 | 2.9 | – | – | – | – | – | – | – | – | – | – | – |
| CRO | N | 3 | – | 8 | – | 16 | – | 4 | – | 2 | – | 1 | – | – | – | – | – | – | – | – | – | – | – | – | – | – | – | – | – | – | – |
|  | Cum. % | 8.8 | – | 32.4 | – | 79.4 | – | 91.2 | – | 97.1 | – | 100 | – | – | – | – | – | – | – | – | – | – | – | – | – | – | – | – | – | – | – |
|  | % | 8.8 | – | 23.5 | – | 47.1 | – | 11.8 | – | 5.9 | – | 2.9 | – | – | – | – | – | – | – | – | – | – | – | – | – | – | – | – | – | – | – |
| CXM | N | – | – | – | – | – | – | – | – | – | 1 | – | – | – | – | 2 | – | 9 | 12 | 6 | 3 | 1 | – | – | – | – | – | – | – | – | – |
|  | Cum. % | – | – | – | – | – | – | – | – | – | 2.9 | – | – | – | – | 8.8 | – | 35.3 | 70.6 | 88.2 | 97.1 | 100 | – | – | – | – | – | – | – | – | – |
|  | % | – | – | – | – | – | – | – | – | – | 2.9 | – | – | – | – | 5.9 | – | 26.5 | 35.3 | 17.6 | 8.8 | 2.9 | – | – | – | – | – | – | – | – | – |
| CLR | N | – | – | – | – | – | – | – | – | – | – | – | – | – | – | – | 2 | – | – | 1 | 1 | 14 | – | 13 | – | 2 | – | 1 | – | – | – |
|  | Cum. % | – | – | – | – | – | – | – | – | – | – | – | – | – | – | – | 5.9 | – | – | 8.8 | 11.8 | 52.9 | – | 91.2 | – | 97.1 | – | 100 | – | – | – |
|  | % | – | – | – | – | – | – | – | – | – | – | – | – | – | – | – | 5.9 | – | – | 2.9 | 2.9 | 41.2 | – | 38.2 | – | 5.9 | – | 2.9 | – | – | – |
| LVX | N | – | – | – | 1 | – | – | 2 | – | 19 | – | 2 | – | – | – | 1 | – | – | 1 | 1 | 3 | – | – | – | 4 | – | – | – | – | – | – |
|  | Cum. % | – | – | – | 2.9 | – | – | 8.8 | – | 64.7 | – | 70.6 | – | – | – | 73.5 | – | – | 76.5 | 79.4 | 88.2 | – | – | – | 100 | – | – | – | – | – | – |
|  | % | – | – | – | 2.9 | – | – | 5.9 | – | 55.9 | – | 5.9 | – | – | – | 2.9 | – | – | 2.9 | 2.9 | 8.8 | – | – | – | 11.8 | – | – | – | – | – | – |
| MXF | N | – | – | – | 1 | – | – | 4 | – | 15 | – | 4 | – | – | – | 1 | – | – | 2 | 1 | 1 | 1 | – | 3 | 1 | – | – | – | – | – | – |
|  | Cum. % | – | – | – | 2.9 | – | – | 14.7 | – | 58.8 | – | 70.6 | – | – | – | 73.5 | – | – | 79.4 | 82.4 | 85.3 | 88.2 | – | 97.1 | 100 | – | – | – | – | – | – |
|  | % | – | – | – | 2.9 | – | – | 11.8 | – | 44.1 | – | 11.8 | – | – | – | 2.9 | – | – | 5.9 | 2.9 | 2.9 | 2.9 | – | 8.8 | 2.9 | – | – | – | – | – | – |
| TET | N | – | – | – | – | – | – | – | – | – | – | – | – | – | – | – | – | 15 | 17 | – | – | – | – | 1 | – | 1 | – | – | – | – | – |
|  | Cum. % | – | – | – | – | – | – | – | – | – | – | – | – | – | – | – | – | 44.1 | 94.1 | – | – | – | – | 97.1 | – | 100 | – | – | – | – | – |
|  | % | – | – | – | – | – | – | – | – | – | – | – | – | – | – | – | – | 44.1 | 50.0 | – | – | – | – | 2.9 | – | 2.9 | – | – | – | – | – |
| SXT | N | – | – | – | – | – | 1 | – | – | – | – | – | – | 4 | – | 2 | – | 6 | 3 | 2 | 1 | 3 | – | 5 | 7 | – | – | – | – | – | – |
|  | Cum. % | – | – | – | – | – | 2.9 | – | – | – | – | – | – | 14.7 | – | 20.6 | – | 38.2 | 47.1 | 52.9 | 55.9 | 64.7 | – | 79.4 | 100 | – | – | – | – | – | – |
|  | % | – | – | – | – | – | 2.9 | – | – | – | – | – | – | 11.8 | – | 5.9 | – | 17.6 | 8.8 | 5.9 | 2.9 | 8.8 | – | 14.7 | 20.6 | – | – | – | – | – | – |

–, not applicable; AMC, amoxicillin/clavulanic acid; AMP, ampicillin; AMX, amoxicillin; AZM, azithromycin; CDN, cefditoren; CEC, cefaclor; CFM, cefixime; CLR, clarithromycin; CPD, cefpodoxime; CRO, ceftriaxone; CTB, ceftibuten; CTX, cefotaxime; Cum., cumulative; CXM, cefuroxime; LVX, levofloxacin; MXF, moxifloxacin; SXT, trimethoprim/sulfamethoxazole; TET, tetracycline.

Bold vertical bars in table correspond to the CLSI-susceptible breakpoints.
